# Exploring Potential Signals of Selection for Disordered Residues in Prokaryotic and Eukaryotic Proteins

**DOI:** 10.1016/j.gpb.2020.06.005

**Published:** 2020-12-18

**Authors:** Arup Panda, Tamir Tuller

**Affiliations:** Department of Biomedical Engineering, Tel Aviv University, Tel Aviv 69978, Israel

**Keywords:** Intrinsically disordered protein, Comparative genomics, Gene function, Proteome evolution, Z-score

## Abstract

**Intrinsically disordered proteins** (IDPs) are an important class of proteins in all domains of life for their functional importance. However, how nature has shaped the disorder potential of prokaryotic and eukaryotic proteins is still not clearly known. Randomly generated sequences are free of any selective constraints, thus these sequences are commonly used as null models. Considering different types of random protein models, here we seek to understand how the disorder potential of natural eukaryotic and prokaryotic proteins differs from random sequences. Comparing proteome-wide disorder content between real and random sequences of 12 model organisms, we noticed that eukaryotic proteins are enriched in disordered regions compared to random sequences, but in prokaryotes such regions are depleted. By analyzing the position-wise disorder profile, we show that there is a generally higher disorder near the N- and C-terminal regions of eukaryotic proteins as compared to the random models; however, either no or a weak such trend was found in prokaryotic proteins. Moreover, here we show that this preference is not caused by the amino acid or nucleotide composition at the respective sites. Instead, these regions were found to be endowed with a higher fraction of protein–protein binding sites, suggesting their functional importance. We discuss several possible explanations for this pattern, such as improving the efficiency of protein–protein interaction, ribosome movement during translation, and post-translational modification*.* However, further studies are needed to clearly understand the biophysical mechanisms causing the trend.

## Introduction

Until the early 1990s, molecular biology studies have mainly focused on globular proteins, with the view that protein function is inherently encoded in its folded 3D structure. However, recent studies suggest that a large number of naturally occurring proteins do not fold into specific 3D structures in their native states [1], [2], [3], [4], [5], [6]. These proteins are commonly known as intrinsically disordered proteins (IDPs) or intrinsically unstructured proteins (IUPs).

IDPs follow unique sets of biophysical characteristics that are very distinct from those of well-structured globular proteins. At the primary structure level, IDPs are enriched by the presence of numerous uncompensated charged groups resulting in a low mean hydrophobicity and a high net charge at neutral pH [1], [2], [3], [4], [5], [6]. Disordered regions are encoded mainly by polar and charged amino acids (specifically, G, R, Q, S, E, and K) and are devoid of hydrophobic and aromatic amino acids [1], [2], [3], [4], [5]. Due to the relatively higher rates of amino acid substitutions and fixation of insertions and deletions, disordered regions are known to evolve at significantly higher rates than ordered regions [7], [8], [9], [10].

Despite their unordered structures, IDPs play central roles in several biological processes [4], [5], [6]. IDPs can complement the functions of globular proteins and carry out several functions that can’t be achieved by globular proteins [4], [5], [6]. Specifically, IDPs play significant roles in signaling and transcription, and in regulatory processes such as control of cell division, apoptosis, and post-translational modifications (PTMs) [4], [5], [6]. Due to their inherent structural flexibility, IDPs can bind a large number of partner proteins [1], [4], [5], [6]. Thus, IDPs can provide the structural basis for binding promiscuity of hub proteins (proteins that bind multiple partners in protein–protein interaction networks) [5], [6], [9]. IDPs often act as flexible linkers between globular domains to facilitate their binding diversity. Another important feature of IDPs is that many of these proteins can undergo a coupled folding and binding process, *i.e*., they can adopt stable secondary structures upon binding with partner molecules [4], [5], [6]. Binding of IDPs with their partner molecules may also be mediated by short motifs known as molecular recognition features (MoRFs) [2], [5], [6], [11], [12]. Computational predictions suggest that IDPs, in general, are highly enriched with MoRFs, indicating their high interaction promiscuity [5], [6], [11], [12].

Several previous initiatives attempted to estimate the abundance of IDPs in the different domains of life. These studies suggest that disordered residues, in general, are more prevalent in complex organisms such as multi-cellular eukaryotes than in unicellular bacterial and archaeal organisms [13], [14], [15]. Disordered residues appear to help complex organisms sustain their functional and regulatory complexities [13]. IDPs also play significant roles in the evolution of various prokaryotic and eukaryotic organisms [16], [17].

The sequences found in nature are considered to be only a small subset of all possible sequences, refined and edited over millions of years of evolutionary constraints [18]. Randomly generated artificial sequences provide an important tool to understand the direction of this refinement. Natural sequences evolve under constraints imposed by their structural and functional requirements [18]. Random DNA sequences, being free from such pressures, are widely used as null models to explore the extent of selection on different traits on naturally occurring protein and DNA sequences [19], [20]. Random DNA sequences are often used for exploring the evolutionary signatures that discriminate real sequences from random ones [18], [21]. Further, random sequences provide important insights regarding the structural and functional basis of extant protein and DNA sequences [22], [18], [19], [20].

Previous studies attempted to understand how the disorder potential of naturally occurring proteins differs from that of randomly generated sequences [23], [24]. Understanding how the disorder potential of natural protein sequences differs from that of random sequences could provide insights into their evolutionary history. However, as these studies analyzed the disorder level of complete proteins, there is no clear understanding of the regions in the proteins that might be under evolutionary pressure for strong or weak folding and what their functional implications are. Therefore, in this study, we explore these crucial aspects. Our major objectives are twofold: to test whether there is any preference or avoidance of disordered residues in naturally occurring eukaryotic and prokaryotic proteins as compared to random expectations and to find whether there is any site-specific variation in this preference for disordered residues along the protein length.

**Figure 1:**
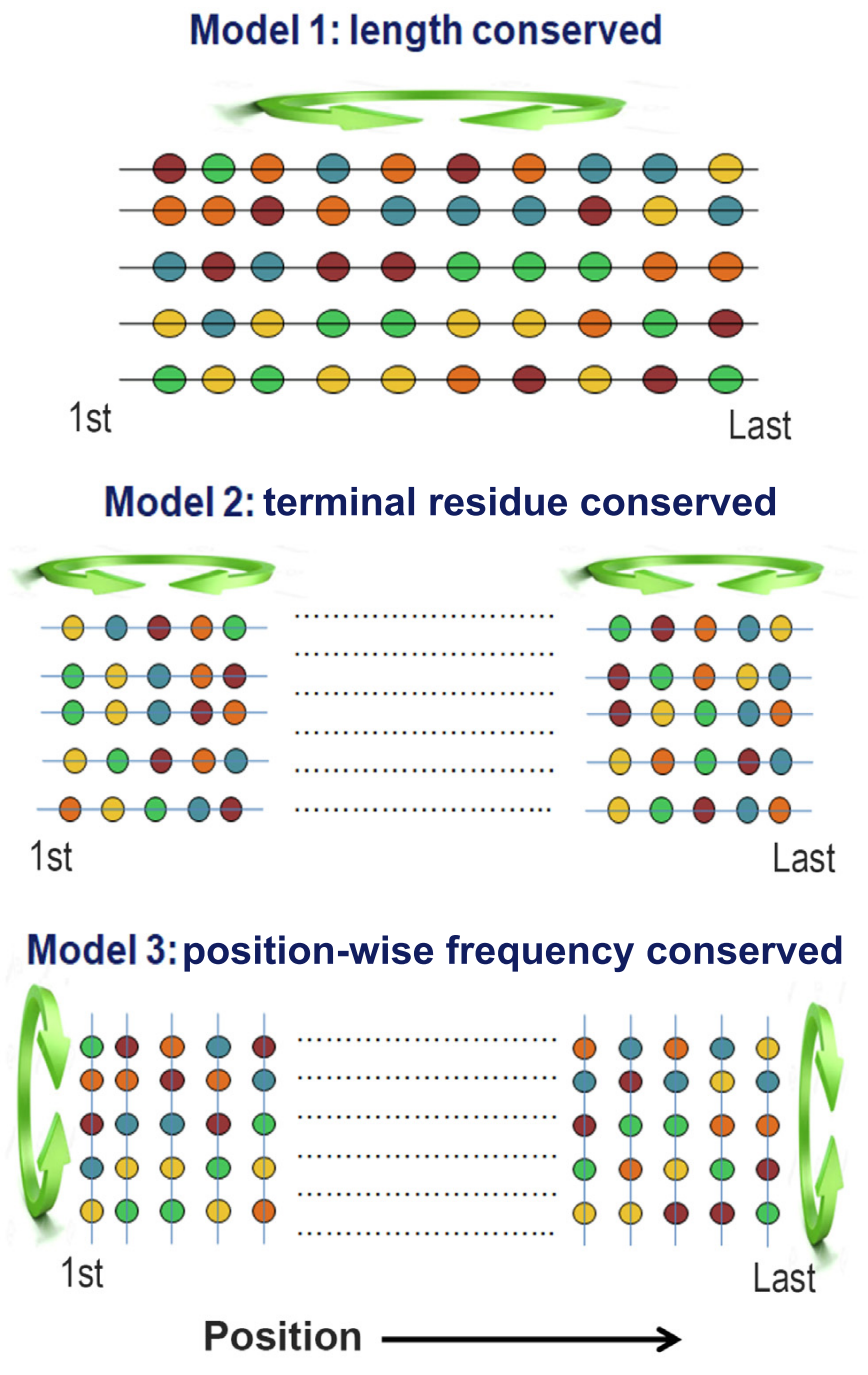
**Generation of random protein models** For each naturally occurring protein sequence, we generated three randomized variants. Model 1: Random shuffling of amino acids of each real protein. This random model, designated as length conserved random model, maintains overall amino acid composition and length of each real protein. Model 2: Shuffling of amino acids (up to the first 200 and last 200 positions) at the N- and C-termini of each real protein. This random model was designated as terminal residue conserved random model which preserves the amino composition of the respective terminal regions. Model 3: Shuffling of amino acids in each position of real proteins. For this random model (position-wise frequency conserved / column-wise random model), naturally occurring proteins of each organism were aligned from both ends and then shuffled position-wise. This model preserves the overall amino acid frequencies at each position of the alignment (see main text for details).

To test these aspects, we generated three kinds of random protein models: 1) that preserves the fundamental properties of real proteins such as their overall amino acid frequencies and length, 2) that preserves the characteristics of terminal regions, and 3) that preserves position-wise amino acid frequencies at each position of the naturally occurring proteins (Figure 1). The order or disorder status of both real and random protein models was predicted based on the mutual agreement among four disorder prediction algorithms. We also verified all the major results with an additional set of prediction algorithms using a majority-vote consensus approach. To understand the evolutionary trend, we first compared the overall disorder propensities of real and random proteins of each species and then examined their disorder scores position-wise. Both approaches suggest that naturally occurring eukaryotic proteins contain a higher percentage of disordered residues (here referred to as protein disorder content) compared to the corresponding random sequences and this preference is more pronounced at the terminal regions of eukaryotic proteins than the other regions. Considering several factors that may explain this trend, we argue that this is independent of selection for any other traits. Then, we emphasized the functional significance of the observed trends. We believe that our study will advance understanding of the forces shaping the disorder propensities of protein sequences.

## Results

### Eukaryotic and prokaryotic proteins have different disorder propensities

Comparing the disorder content of 10,000 real and randomly generated proteins, it was previously shown that real proteins are more disordered than random sequences [23]. However as that study combined sequences from different organisms together, it is not known if all organisms follow similar trends or if there is variation between species. In this study, we systematically compared protein disorder content between real and random sequences species-wise. For each organism, we separately generated random artificial protein sequences preserving the overall amino acid frequencies and length of its real sequences (length conserved random sequences). We considered two measures of protein disorder content, the percentage of all predicted disordered residues and the percentage of disordered residues within long disordered segments, and compared real and random sequences of each test species. These two measures of protein disorder content generally showed similar distributions in the tested species, except in a few cases.

**Figure 2:**
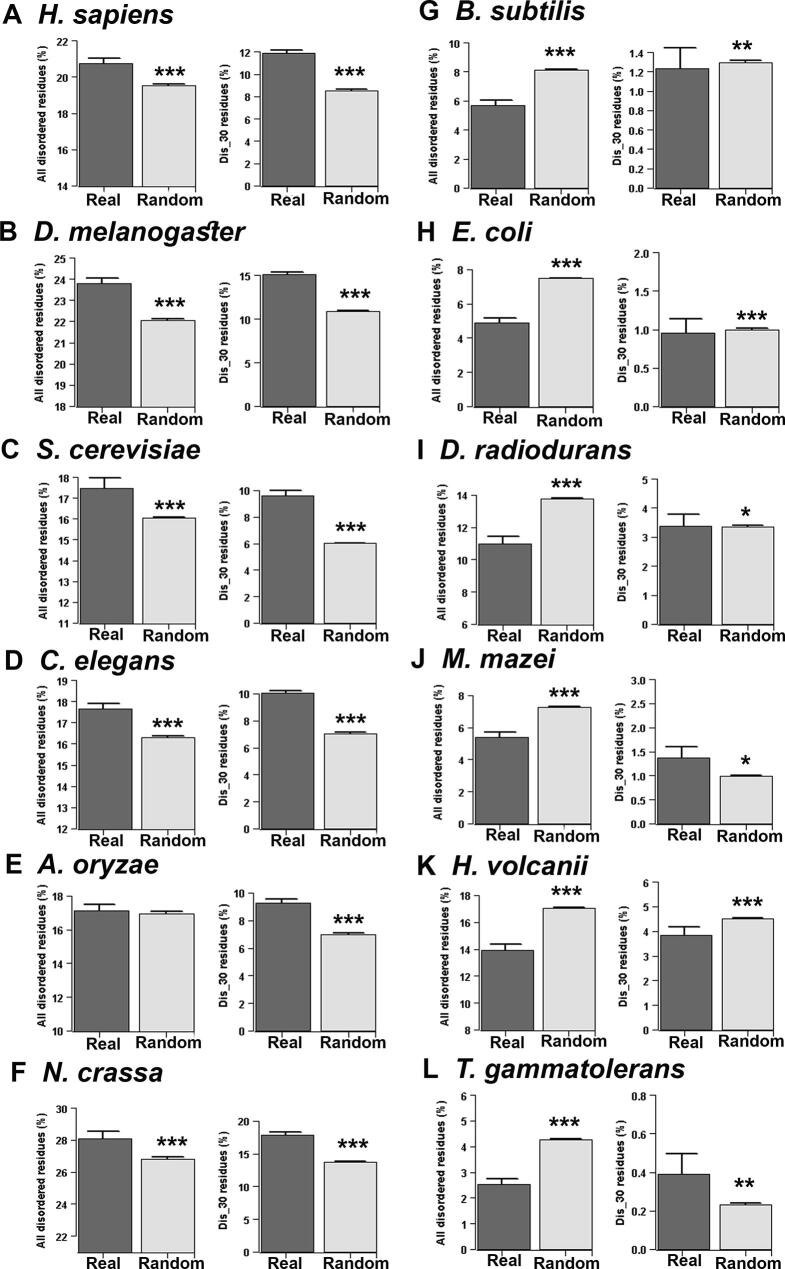
Proteome-wise comparison of disorder content between real and random sequences predicted by consensus approach 1 Graphs show the average disorder content of real and random proteins in six eukaryotes (**A–F**) and six prokaryotes (**G–L**). A. *Homo sapiens*. B. *Drosophila melanogaster*. C. *Saccharomyces cerevisiae*. D. *Caenorhabditis elegans*. E. *Aspergillus oryzae*. F. *Neurospora crassa*. G. *Bacillus subtilis*. H. *Escherichia coli*. I. *Deinococcus radiodurans*. J. *Methanosarcina mazei*. K. *Haloferax volcanii*. L. *Thermococcus gammatolerans*. Disorder content is calculated as the percentage of disordered residues in each protein (predicted by the consensus approach 1, see main text for details), and then averaged over all the proteins in each group. Disorder content is calculated considering all predicted disordered residues (denoted as percentage of all disordered residues) and considering disordered residues only in long disordered regions (30 or more consecutive disordered residues; denoted as percentage of Dis_30 residues). So, there are two plots in each panel, showing the proportion of disordered residues of both real and random sequences calculated by these two approaches. *P* values are calculated by Mann–Whitney *U* test by comparing disorder content between real and random sequences of each species. Significant difference between real and random sequences is shown with *, *P* < 0.05; **, *P* < 1 × 10^−4^; ***, *P* < 1 × 10^−6^. Error bars show standard error at 95% confidence interval. The numbers of proteins in the real datasets are as follows: *H. sapiens*, 16,384; *D. melanogaster*, 24,799; *C. elegans*, 21,187; *S. cerevisiae*, 4772; *A. oryzae*, 9830; *N. crassa*, 8899; *B. subtilis*, 2588; *E. coli*, 2838; *D. radiodurans*, 2080; *M. mazei*, 2063; *H. volcanii*, 2410; *T. gammatolerans*, 1346. In each organism, the number of proteins in the random dataset is 10 times than the real dataset.

**Supplementary figure S1:**
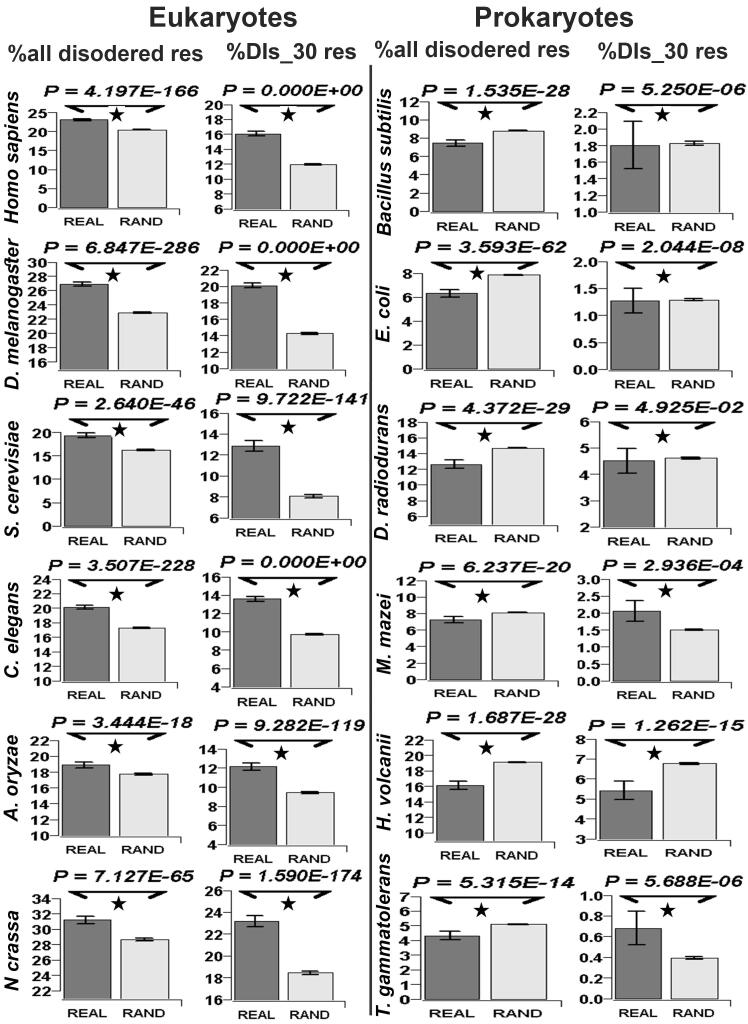
**Proteome-wise comparison of disorder content between real and random sequences predicted by consensus approach 2**. The graph shows the average disorder content of real and random proteins of each of the test species. Disordered content is calculated as the percentage of disordered residues in each protein (predicted by the consensus approach 2, see main text for details) then averaged over all the proteins in each group (real or random). Disordered content is calculated considering all predicted disordered residues (denoted as percentages of all disordered residues) and considering disordered residues only in long disordered regions (30 or more consecutive disordered residues) (denoted as % of DIS_30 residues). So, there are two panels for each species, each panel showing the proportion of disordered residues between real and random sequences calculated by these two approaches. *P* values were calculated by Mann–Whitney *U* test comparing disordered content between real and random sequences of each species. Here error bars show standard error at 95% confidence interval. Significant difference (*P* < 0.05) was shown with *. Here *P* stands for the *P* values obtained by Mann–Whitney *U* test.

Proteome-wide average disorder content of real and random sequences of each species is shown in Figure 2. In contrast to an earlier report of high protein disorder among real sequences [23], we found that natural sequences can have more or less disorder content compared to the random sequences depending on the characteristics of the species. Specifically, in eukaryotes (*Homo sapiens*, *Drosophila melanogaster*, *Caenorhabditis elegans*, *Saccharomyces cerevisiae*, *Aspergillus oryzae*, and *Neurospora crassa*), real sequences were found to be more disordered, while in prokaryotes (*Bacillus subtilis*, *Escherichia coli*, *Deinococcus radiodurans*, *Methanosarcina mazei*, *Haloferax volcanii*, and *Thermococcus gammatolerans*), real sequences were found to be less disordered than their corresponding random sequences (Figure 2). These results suggested a general trend that in eukaryotes natural sequences are more disordered (in terms of percentage of all predicted disordered residues and percentage of disordered residues within long disordered segments) than their corresponding random sequences, while prokaryotes follow opposite behavior (Figure 2). We validated these results with another set of prediction algorithms (see Materials and methods, consensus approach 2) and found similar overall results (Figure S1). Thus the earlier report of high protein disorder among naturally occurring sequences [23] agrees with our observations in eukaryotes but not in prokaryotes.

**Supplementary figure S2:**
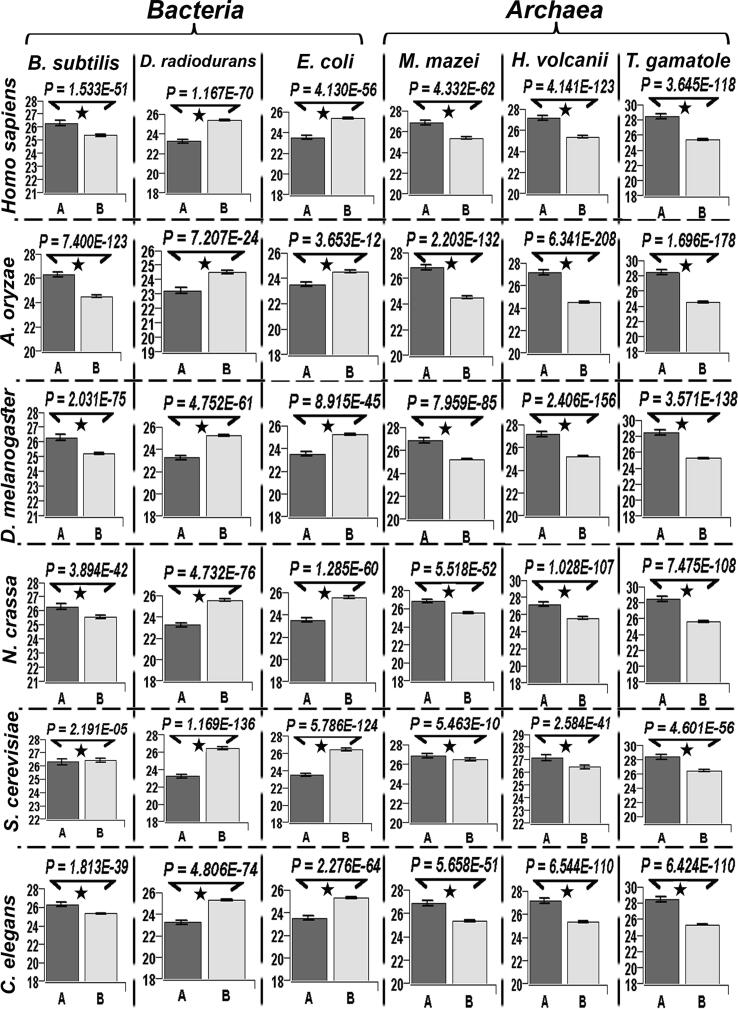
**Species to species comparison of the distribution of charged residues between the test prokaryotic and eukaryotic organisms**. The graph shows the average % of charged residues in the proteomes of our test organisms. Here we compared the proportion of charged residues of each eukaryotic species (denoted as species B) with that of each prokaryotic species (denoted as species A). *P* values were calculated by Mann–Whitney *U* test. Here error bars show standard error at 95% confidence interval. Significant difference (*P* < 0.05) was shown with *. Here *P* stands for the *P* values obtained by Mann–Whitney *U* test.

**Supplementary figure S3:**
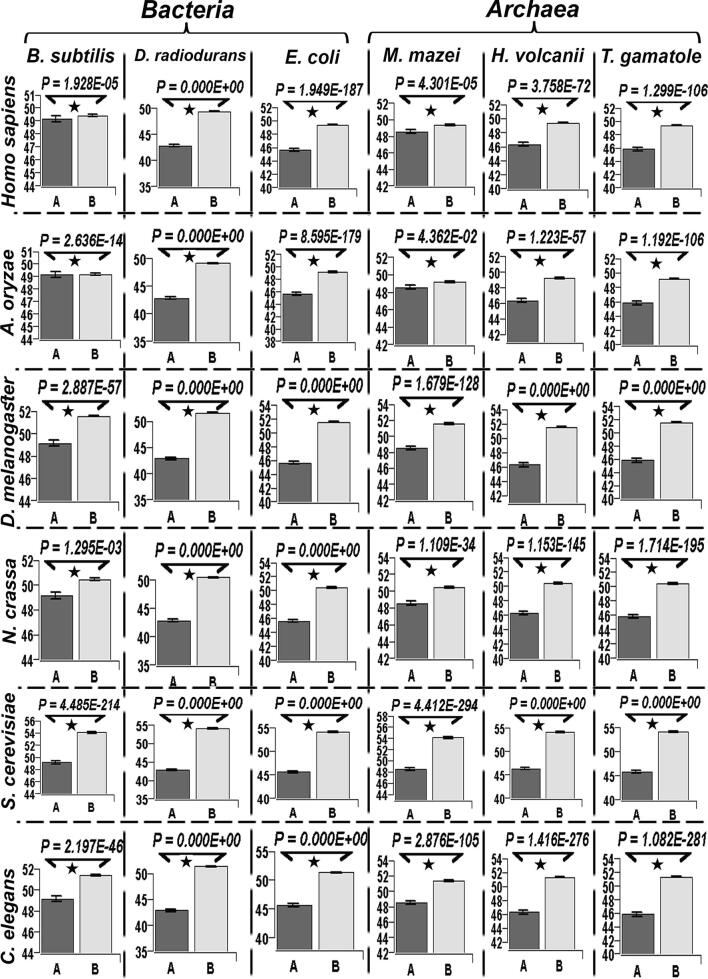
**Species to species comparison of the distribution of polar residues between the test prokaryotic and eukaryotic organisms**. The graph shows the average % of polar residues in the proteomes of our test organisms. Here we compared the proportion of polar residues of each eukaryotic species (denoted as species B) with that of each prokaryotic species (denoted as species A). *P* values were calculated by Mann–Whitney *U* test. Here error bars show standard error at 95% confidence interval. Significant difference (*P* < 0.05) was shown with *. Here *P* stands for the *P* values obtained by Mann–Whitney *U* test.

Disordered regions, in general, are encoded by polar and charged residues [1], [2], [3], therefore proteomes enriched with these types of amino acids are expected to be more disordered. When we compared the proportion of polar (S, T, N, D, E, Q, R, H, K, and Y) and charged (D, E, R, H, and K) residues in the proteomes of the organisms tested here, we did not find any consistent trend in the distributions of charged residues (Figure S2). Instead, our result suggested that prokaryotes may have higher or lower percentages of charged residues compared to eukaryotes. However, we found a distinct pattern in the distribution of polar residues, *i.e.*, the proportion of polar residues is lower in prokaryotic proteomes than in the eukaryotic proteomes considered in this study (Figure S3). Therefore, it may be assumed that the higher proportion of disordered residues in the eukaryotic proteomes is due to their excess polar residues. However, it is worth noting that in each proteome, we compared disorder content of real sequences with the random sequences specifically derived from those real sequences preserving the overall amino acid composition and length of the proteins. Thus these results are not expected to be biased by factors such as amino acid composition or length of the protein.

**Supplementary figure S4:**
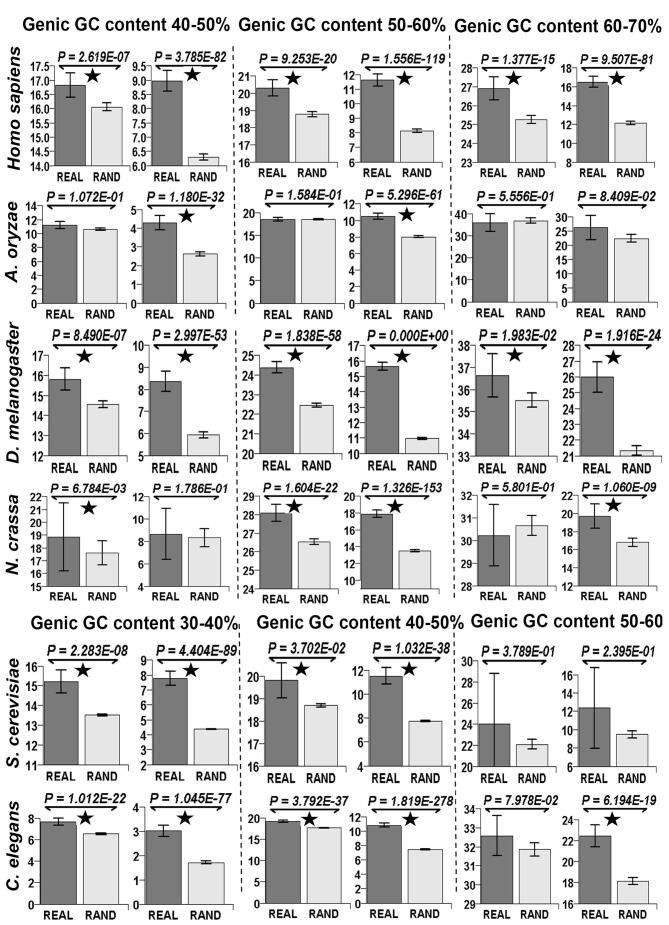
**Comparison of disorder content between real and random sequences of eukaryotic species in GC bins**. In each species, naturally occurring protein sequences were grouped in GC bins according to the GC content of their coding sequences then their disorder content was compared with that of their corresponding length conserved random sequences. In each species, GC bins were defined following distribution of genic GC content in steps of 10% GC variation (see main text). Disorder content of both real and random datasets was calculated by the consensus approach 1 (see main text) considering (*i*) all the predicted disordered residues (denoted as percentages of all disordered residues), and (*ii*) considering disordered residues only in long disordered regions (denoted as % of DIS_30 residues). So, in each GC bin there are two panels, each panel showing the proportion of disordered residues between real and random sequences calculated by these two approaches. *P* values were calculated by Mann–Whitney *U* test comparing disorder content between real and random sequences in each GC bin. Here error bars show standard error at 95% confidence interval. Significant difference (*P* < 0.05) was shown with *. Here *P* stands for the *P* values obtained by Mann–Whitney *U* test.

**Supplementary figure S5:**
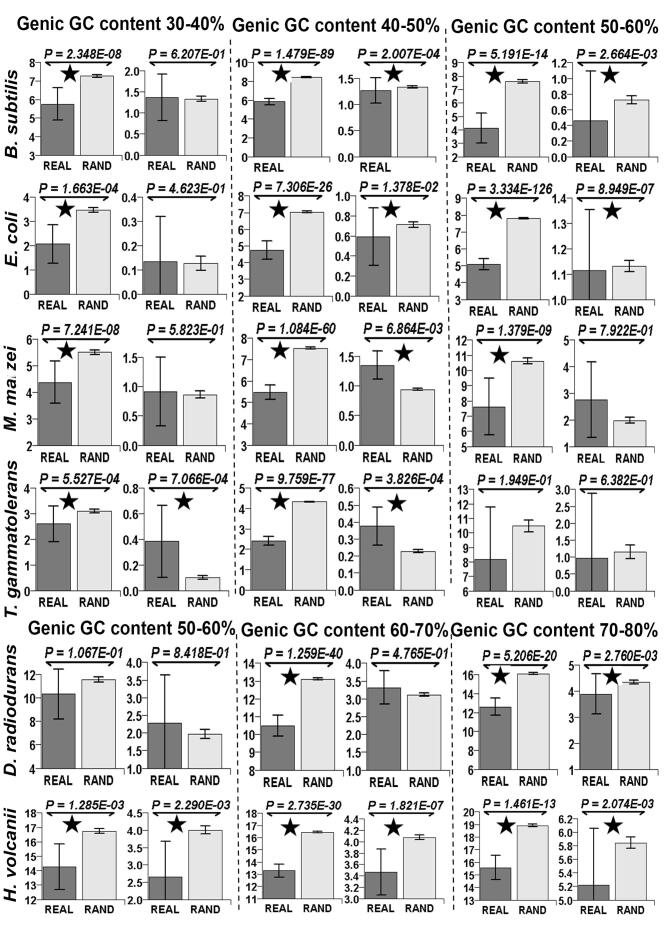
**Comparison of disorder content between real and random sequences of prokaryotic species in GC bins**. In each species, naturally occurring protein sequences were grouped in GC bins according to the GC content of their coding sequences then their disorder content was compared with that of their corresponding length conserved random sequences. In each species, GC bins were defined following distribution of genic GC content in steps of 10% GC variation (see main text). Disorder content of both real and random datasets was calculated by consensus approach 1 (see main text) considering (*i*) all the predicted disordered residues (denoted as percentages of all disordered residues), and (*ii*) considering disordered residues only in long disordered regions (denoted as % of DIS_30 residues). So, in each GC bin there are two panels, each panel showing the proportion of disordered residues between real and random sequences calculated by these two approaches. *P* values were calculated by Mann–Whitney *U* test comparing disordered content between real and random sequences in each GC bin. Here error bars show standard error at 95% confidence interval. Significant difference (*P* < 0.05) was shown with *. Here *P* stands for the *P* value obtained by Mann–Whitney *U* test.

Considering proteins of various genic GC content, Ángyán et al. [24] proposed that structural preferences of real and random proteins strongly depend on the GC content of their protein-coding sequences. To check whether the trend that we observed here depends on the GC content of the coding sequences, we grouped the naturally occurring proteins of each species according to their genic GC content and compared their disorder content with their corresponding length-conserved random sequences. With a few exceptions, we noticed a similar trend as was found considering all proteins without categorization according to GC content (Figures S4 and S5), suggesting that the observed results are independent of the genomic GC content. In general, this study suggests that the previous report of high protein disorder among naturally occurring protein sequences [23] do not apply universally in all organisms; instead, it is evident that prokaryotic and eukaryotic organisms show distinct trends.

### Position-wise enrichment of disorder residues along the protein sequence

To identify the positions that may show a preference for or against disordered residues, we compared the frequency of these residues between natural and random sequences in a position-wise manner (up to the first 150 and last 150 positions). At each position, the disorder propensity of naturally occurring proteins was compared with that of two kinds of random protein models (illustrated in Figure 1): one generated by random permutation of amino acids near each end of every native protein (terminal residue conserved random model) and another generated by permuting the amino acids in each position of the alignment of native proteins in each species (column-wise random model).

Here it is noteworthy that we found bias in the disorder prediction at both terminal regions (discussed in the next section). Therefore, for each position we computed a Z-score which signifies up to what extent disorder scores of real proteins deviates from that of random proteins at that position (in units of standard deviation). A higher Z-score indicates a more statistically significant difference, which is further assessed through *P* values. Since the Z-scores compare disorder scores of real and random proteins as predicted using the same disorder prediction algorithms, it is expected to compensate for the end bias in disorder prediction as the bias for real and random sequences is supposed to be comparable.

**Figure 3:**
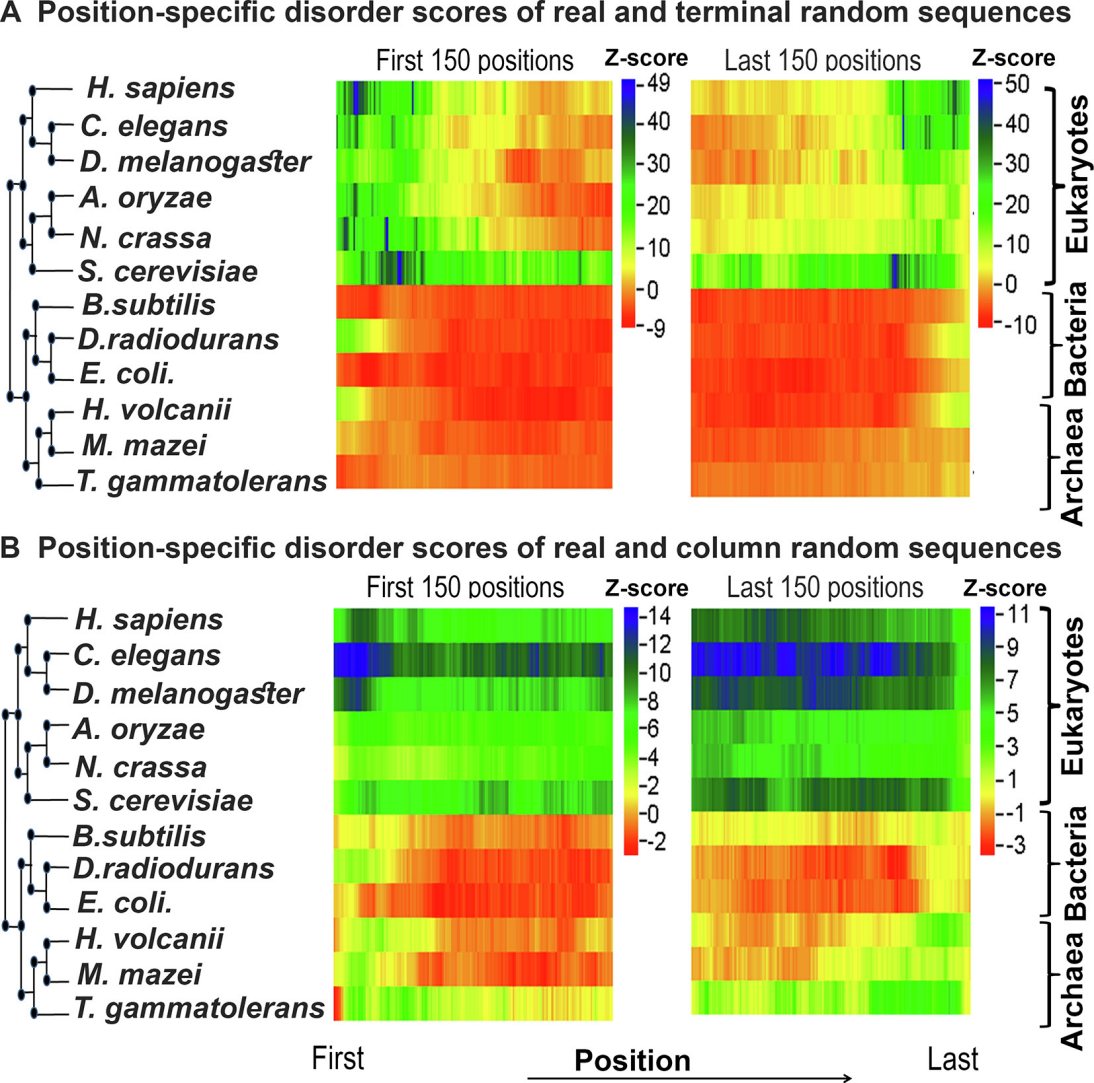
Z-score profile for the position-specific disorder score of each species predicted by consensus approach 1 This figure shows the extent of protein disorder between real and random protein models for the first 150 and last 150 positions of 12 organisms predicted by consensus approach 1 (see main text for details). **A.** Z-scores were calculated by comparing position-specific disorder scores of real and terminal residue conserved random sequences of each species. **B.** Z-scores were calculated by comparing position-specific disorder scores of real and column-wise random sequences of each species. Z-scores were coded in color scheme (color legend). Here positive Z-score indicates enrichment of protein disorder in naturally occurring sequences while negative Z-score indicates the reverse. Organisms are arranged according to their mid-point rooted species tree retrieved from the national center for biotechnology information (NCBI) taxonomic database with the help of their species taxonomic identifier.

**Supplementary figure S6:**
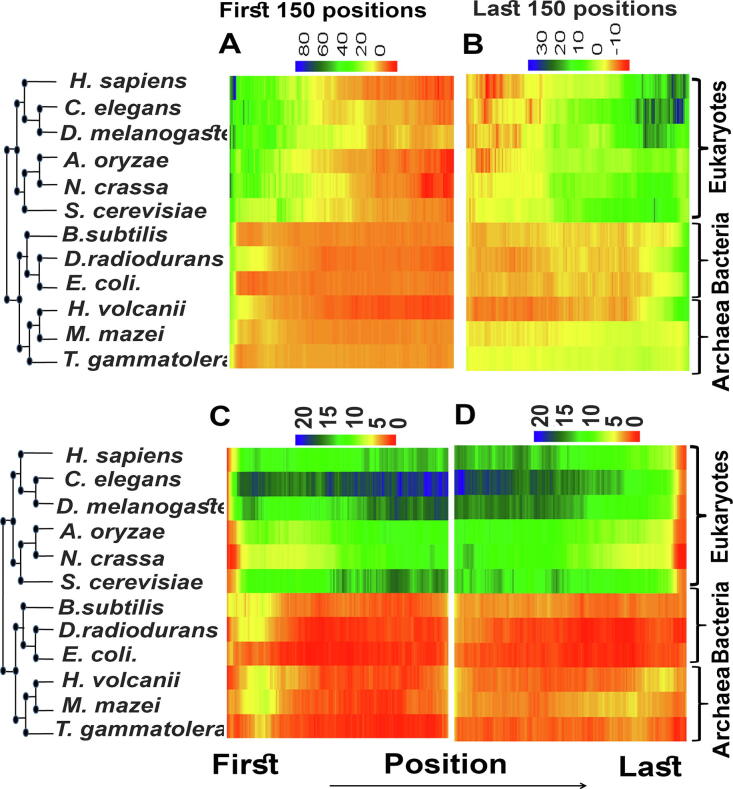
**Z-score profile for the position-specific disorder score of each species predicted by consensus approach 2**. This figure shows the extent of protein disorder between real and random protein models for the first 150 and last 150 positions of 12 organisms considered in this study predicted by consensus approach 2. In panels **A** and **B**, Z-scores were calculated comparing position-specific disorder scores of real and terminal residue conserved random sequences of each species and in panels **C** and **D**, Z-scores were calculated comparing position-specific disorder scores of real and column random sequences of each species. The color scale for each graph is shown on the top of each panel. Here positive Z-score indicates enrichment of protein disorder in naturally occurring sequences while negative Z-score indicates the reverse. Organisms are arranged according to their mid-point rooted species tree retrieved from NCBI taxonomic database with the help of their species taxonomic identifier. NCBI, the national center for biotechnology information.

**Supplementary figure 7:**
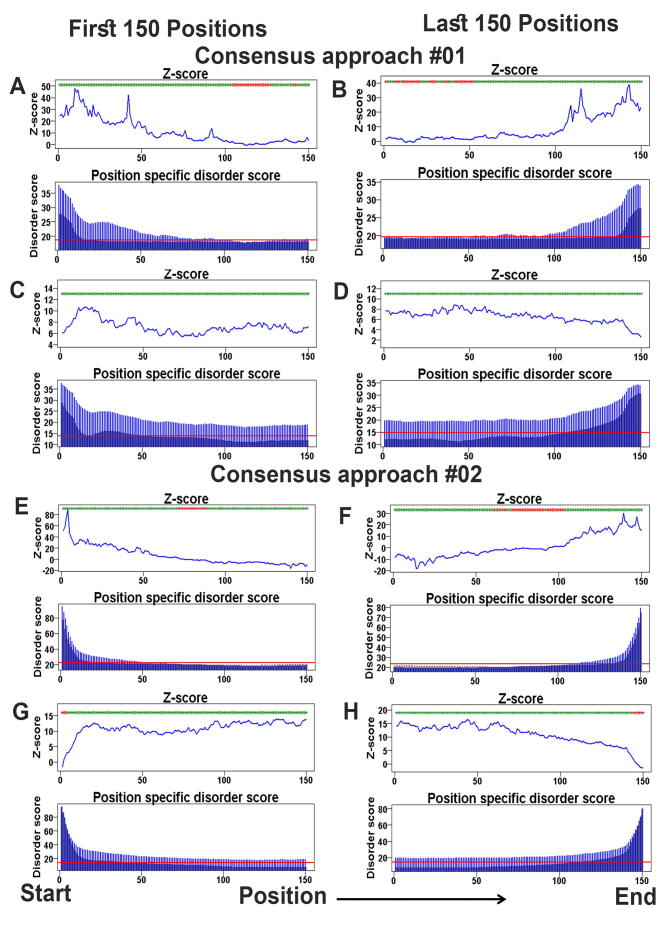
**Z-score profile for position-specific disorder scores of *H. sapiens***. This figure shows the extent of protein disorder between real and random protein models for the first 150 and last 150 positions of human proteins predicted by consensus approach 1 (panels **A**, **B**, **C**, **D**) and consensus approach 2 (panels **E**, **F**, **G**, **H**). Panels A, B, E, F: Comparison of real and terminal residue conserved random sequences. Panels C, D, G, H: Comparison of real and column random sequences. In each panel, vertical blue lines stand for the average position-specific disorder scores of real proteins and gray lines that for random proteins. At each position, the statistical difference between these two measurements is accessed through Z-score (see main text) and is shown with blue line. Corresponding P value at each position is shown either with a green (*P* < 0.05) or red dot (*P* ≥ 0.05) above the Z-score. Here positive Z-score indicates enrichment of protein disorder in naturally occurring sequences while negative Z-score indicates the reverse.

**Supplementary figure S8:**
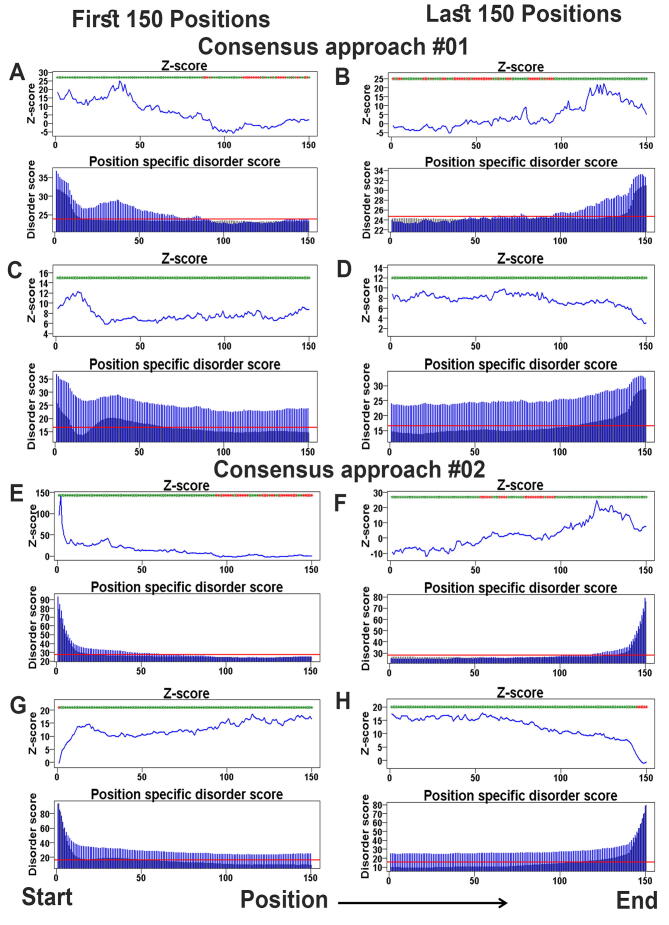
**Z-score profile for position-specific disorder scores of *D. melanogaster***. This figure shows the extent of protein disorder between real and random protein models for the first 150 and last 150 positions of D. melanogaster proteins predicted by consensus approach 1 (panels **A**, **B**, **C**, **D**) and consensus approach 2 (panels **E**, **F**, **G**, **H**). Figure representation is same as Figure S7.

**Supplementary figure S9:**
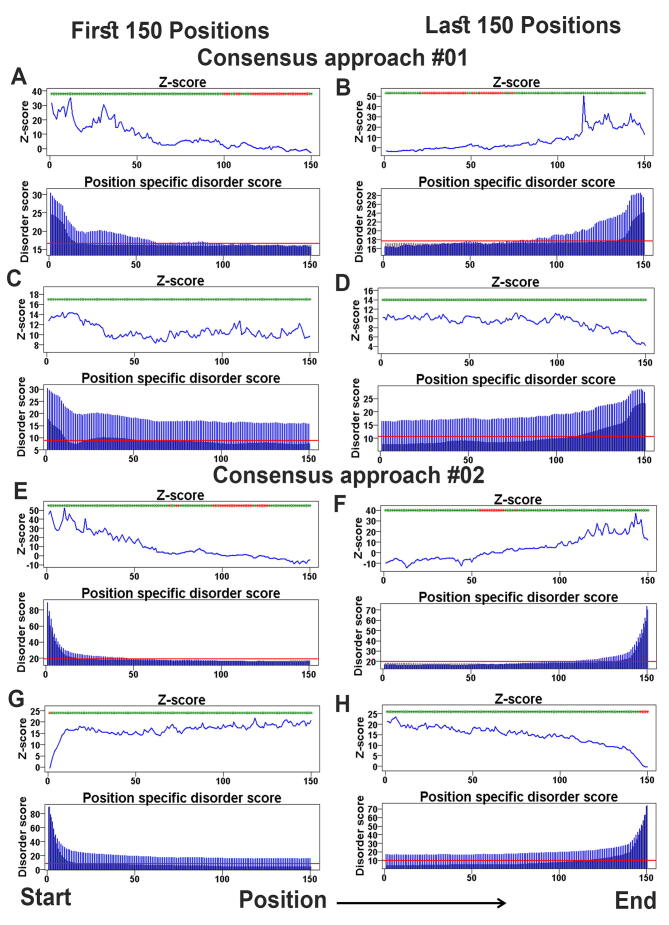
**Z-score profile for position-specific disorder scores of *C. elegans*.** This figure shows the extent of protein disorder between real and random protein models for the first 150 and last 150 positions of *C. elegans* proteins predicted by consensus approach 1 (panels **A**, **B**, **C**, **D**) and consensus approach 2 (panels **E**, **F**, **G**, **H**). Figure representation is same as Figure S7.

**Supplementary figure S10:**
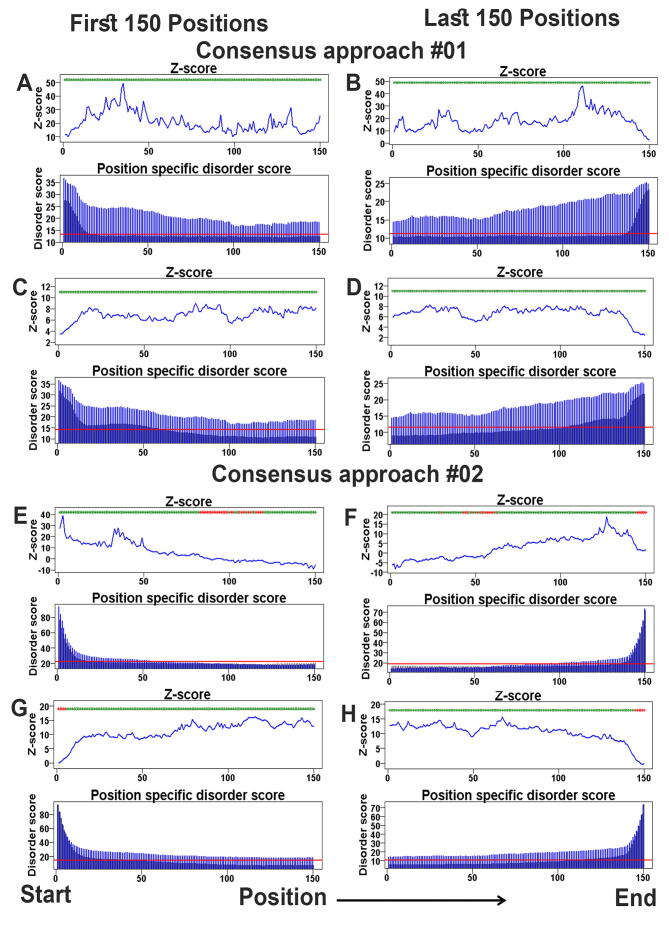
**Z-score profile for position-specific disorder scores of *S. cerevisiae***. This figure shows the extent of protein disorder between real and random protein models for the first 150 and last 150 positions of *S. cerevisiae* proteins predicted by consensus approach 1 (panels **A**, **B**, **C**, **D**) and consensus approach 2 (panels **E**, **F**, **G**, **H**). Figure representation is same as Figure S7.

**Supplementary figure S11:**
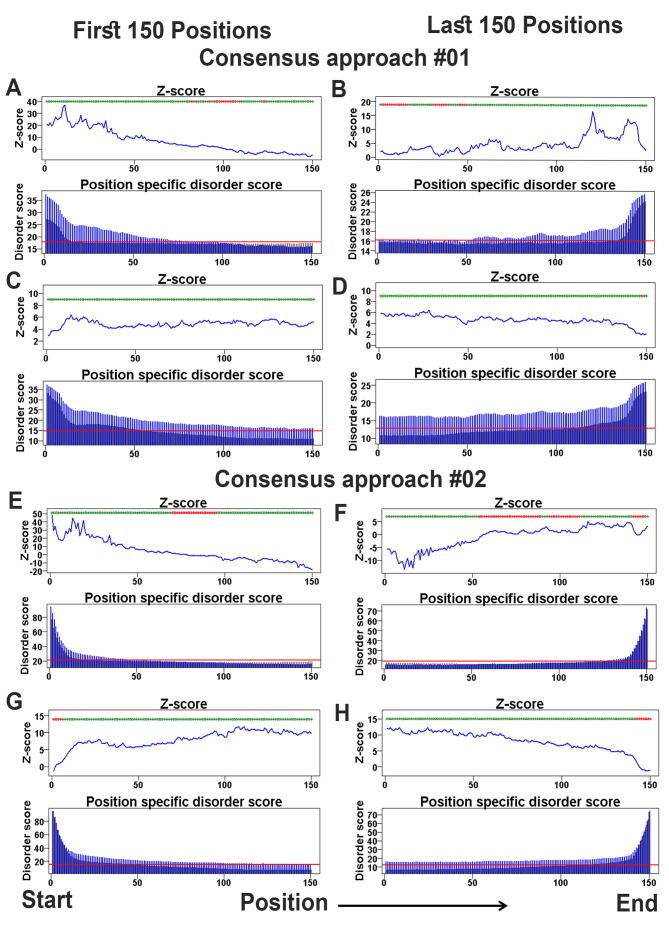
**Z-score profile for position-specific disorder scores of *A. oryzae*.** This figure shows the extent of protein disorder between real and random protein models for the first 150 and last 150 positions of *A. oryzae* proteins predicted by consensus approach 1 (panels **A**, **B**, **C**, **D**) and consensus approach 2 (panels **E**, **F**, **G**, **H**). Figure representation is same as Figure S7.

**Supplementary figure S12:**
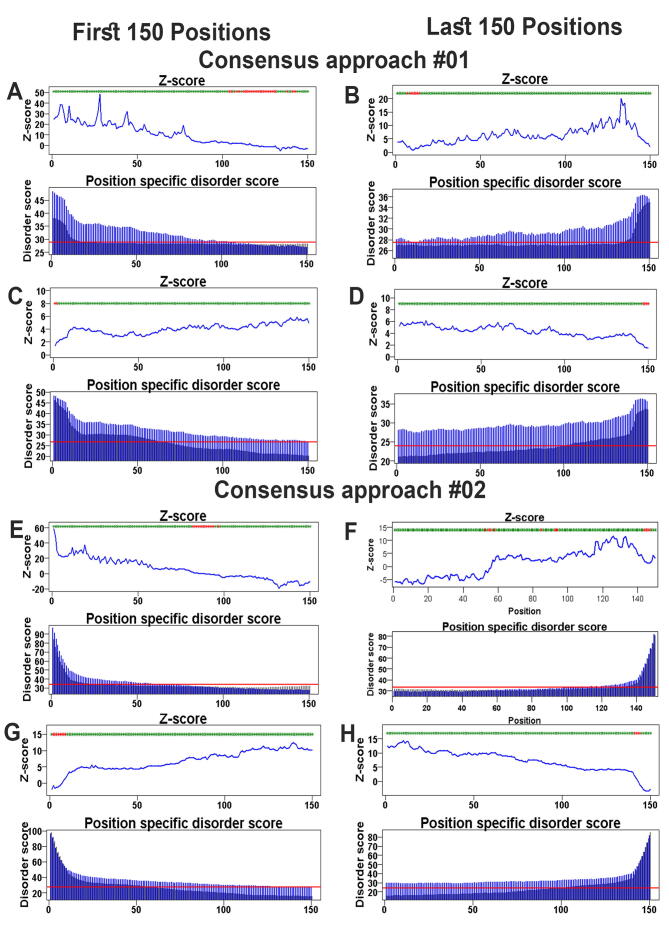
**Z-score profile for position-specific disorder scores of *N. crassa***. This figure shows the extent of protein disorder between real and random protein models for the first 150 and last 150 positions of *N. crassa* proteins predicted by consensus approach 1 (panels **A**, **B**, **C**, **D**) and consensus approach 2 (panels **E**, **F**, **G**, **H**). Figure representation is same as Figure S7.

**Supplementary figure S13:**
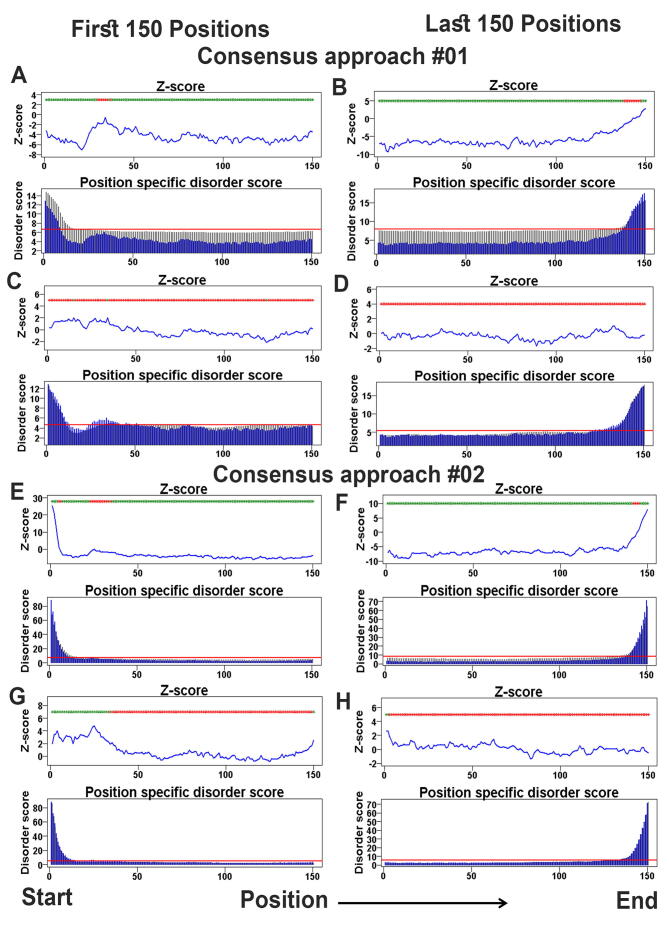
**Z-score profile for position-specific disorder scores of *B. subtilis***. This figure shows the extent of protein disorder between real and random protein models for the first 150 and last 150 positions of *B. subtilis* proteins predicted by consensus approach 1 (panels **A**, **B**, **C**, **D**) and consensus approach 2 (panels **E**, **F**, **G**, **H**). Figure representation is same as Figure S7.

**Supplementary figure S14:**
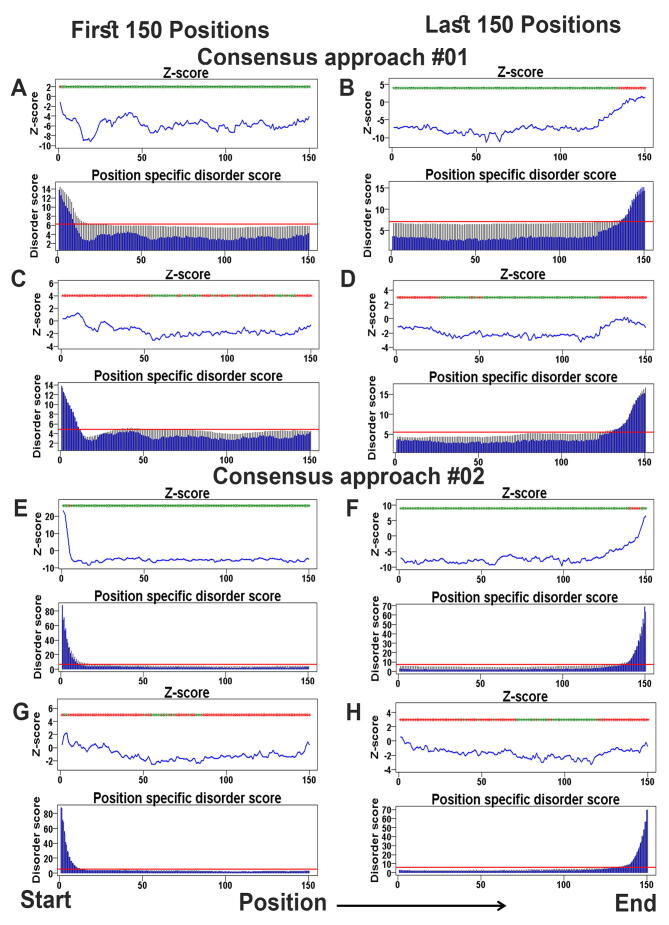
**Z-score profile for position-specific disorder scores of *E. coli***. This figure shows the extent of protein disorder between real and random protein models for the first 150 and last 150 positions of *E. coli* proteins predicted by consensus approach 1 (panels **A**, **B**, **C**, **D**) and consensus approach 2 (panels **E**, **F**, **G**, **H**). Figure representation is same as Figure S7.

**Supplementary figure S15:**
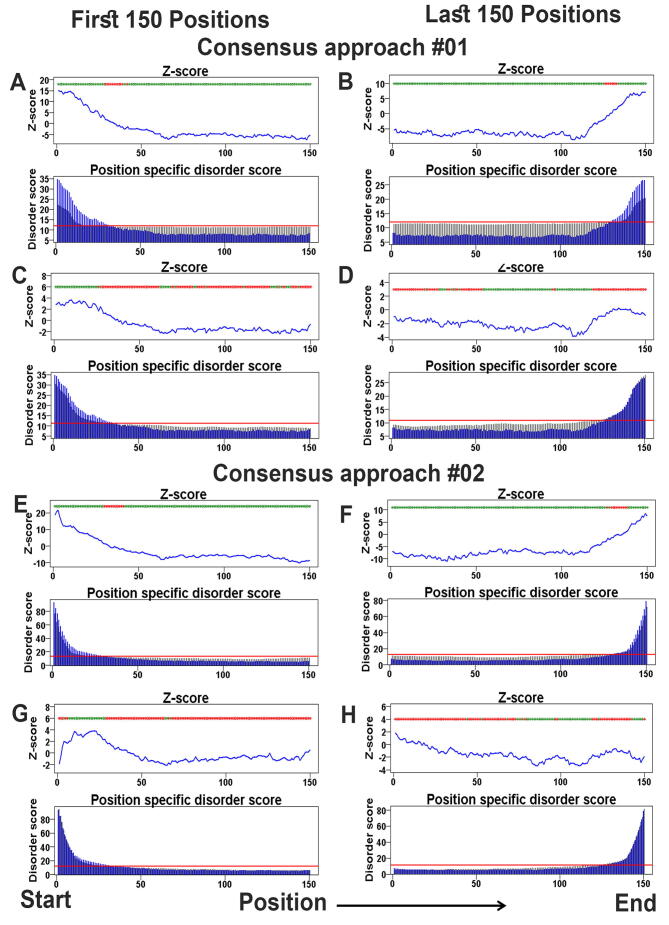
**Z-score profile for position-specific disorder scores of *D. radiodurans***. This figure shows the extent of protein disorder between real and random protein models for the first 150 and last 150 positions of *D. radiodurans* proteins predicted by consensus approach 1 (panels **A**, **B**, **C**, **D**) and consensus approach 2 (panels **E**, **F**, **G**, **H**). Figure representation is same as Figure S7.

**Supplementary figure S16:**
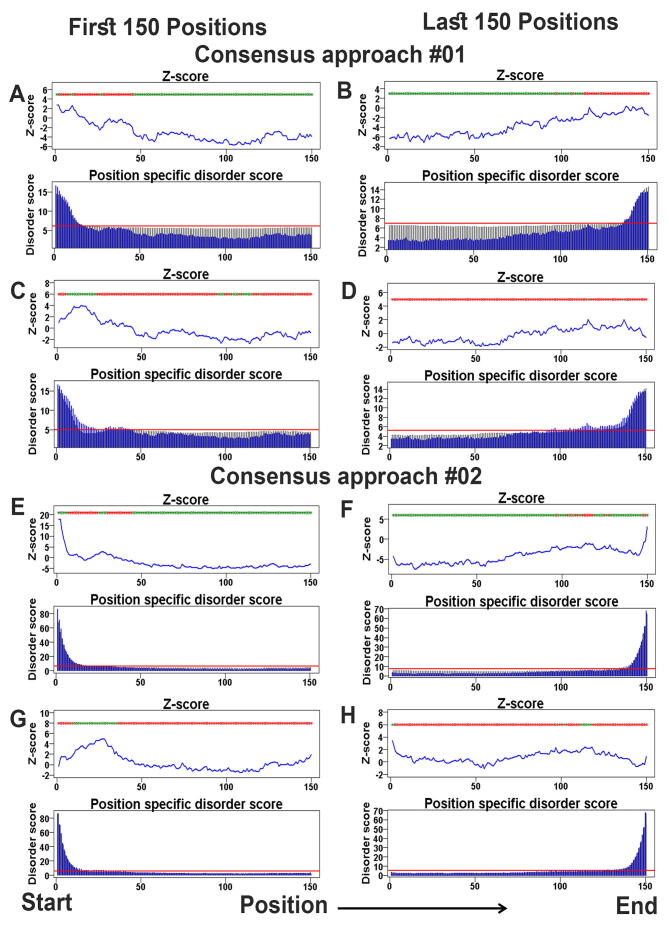
**Z-score profile for position-specific disorder scores of *M. mazei***. This figure shows the extent of protein disorder between real and random protein models for the first 150 and last 150 positions of *M. mazei* proteins predicted by consensus approach 1 (panels **A**, **B**, **C**, **D**) and consensus approach 2 (panels **E**, **F**, **G**, **H**). Figure representation is same as Figure S7.

**Supplementary figure S17:**
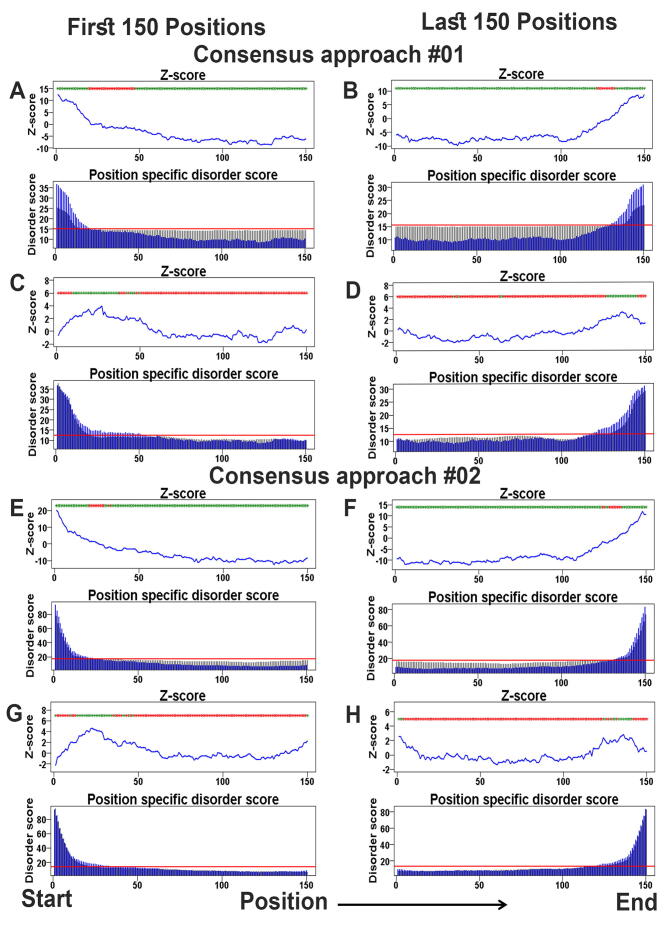
**Z-score profile for position-specific disorder scores of *H. volcanii***. This figure shows the extent of protein disorder between real and random protein models for the first 150 and last 150 positions of *H. volcanii* proteins predicted by consensus approach 1 (panels **A**, **B**, **C**, **D**) and consensus approach 2 (panels **E**, **F**, **G**, **H**). Figure representation is same as Figure S7.

**Supplementary figure S18:**
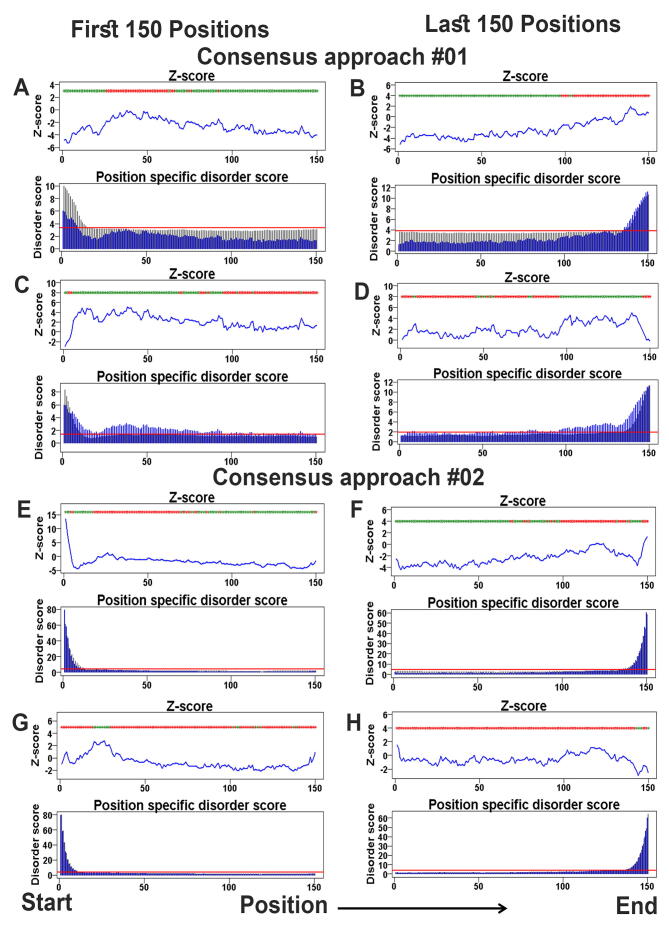
**Z-score profile for position-specific disorder scores of *T. gammatolerans***. This figure shows the extent of protein disorder between real and random protein models for the first 150 and last 150 positions of *T. gammatolerans* proteins predicted by consensus approach 1 (panels **A**, **B**, **C**, **D**) and consensus approach 2 (panels **E**, **F**, **G**, **H**). Figure representation is same as Figure S7.

Position-wise Z-score profiles of the 12 species considered in this study are shown in Figure 3 (by consensus approach 1) and Figure S6 (by consensus approach 2). Detailed graphs for each species can be found in Figures S7–S18, which show the position-specific disorder profile for the first and last 150 positions in the native proteins of each species and their random variants predicted by consensus approach 1 (panels A–D) and consensus approach 2 (panels E–H), respectively. The results based on the terminal residue conserved random model showed that in eukaryotes there is a clear increase in protein disorder (associated with high positive Z-scores) up to the first 100 and last 100 residues of naturally occurring proteins when compared with the terminal residue conserved random proteins (Figure 3A, Figure S6A and B, panels A, B, E, and F in Figures S7–S12). The trend of higher disorder seems to be stronger near the N-terminal regions than near the C-terminal regions (Figure 3A). In contrast, in most of the prokaryotes studied here, we noticed significantly lower disorder scores (negative or weak positive Z-scores) near the terminal regions of native sequences when compared with the same random model (Figure 3A, Figure S6A and B, panels A, B, E, and F in Figures S13, S14, S16, and S18). However, in *D. radiodurans* (Figure S15A, B, E, and F) and *H. volcanii* (Figure S17A, B, E, and F) we found weak positive Z-scores up to the first 10–15 and last 10–15 residues. These results indicated that there is a clear enrichment of disordered residues near the terminal regions of eukaryotic proteins. In prokaryotes, however, this effect is either weak or not observed.

Considering another random model, which preserves position-wise amino acid composition (column-wise random sequences), we observed that in eukaryotes natural sequences have a higher proportion of disordered residues (positive but more or less similar Z-scores) than random sequences throughout the considered regions (Figure 3B, Figure S6C and D, panels C, D, G, and H in Figures S7–S12). However, in prokaryotes, we did not find such significant differences in the disorder score between real and column-wise random sequences near the terminal regions (Figure 3B, Figure S6C and D, panels C, D, G, and H in Figures S13–S18). To ensure that these trends are not the artifacts of the disorder prediction algorithms used in this study, we repeated these tests with an additional set of disorder prediction algorithms (consensus approach 2). Using this approach, we found similar results in general (Figures S7–S18, panels E, F, G, and H), except at the very extreme ends of the proteins. At the extreme ends, up to the first 5–6 and last 5–6 residues at the most, we found lower Z-scores compared to the results obtained by our consensus approach 1.

**Supplementary figure S19:**
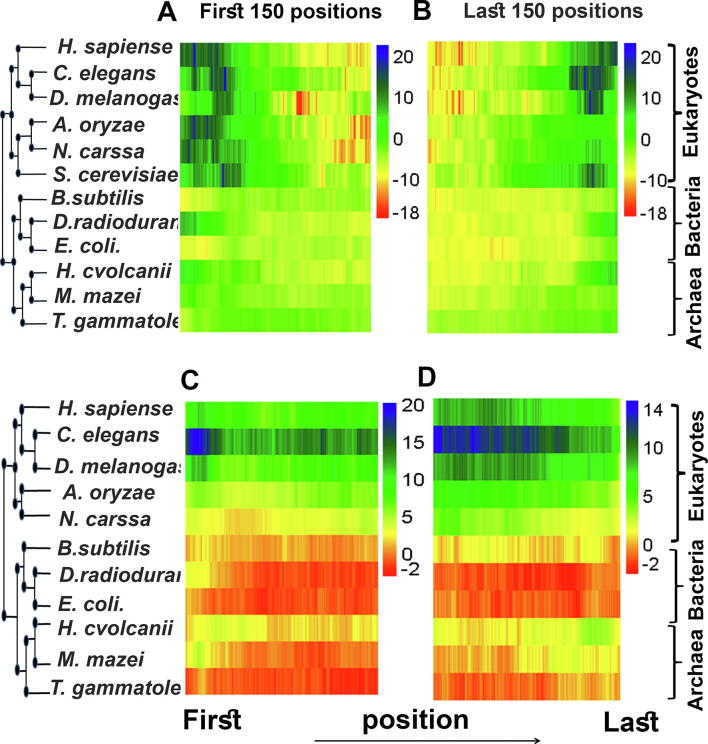
**Z-score profile for position-specific disorder scores predicted by IUPred2A (version long)**. This figure shows the extent of protein disorder between real and random protein models for the first 150 and last 150 positions of 12 organisms considered in this study predicted by IUPred2A algorithm (version long). **A**. and **B**.: comparison of real and terminal residue conserved random sequences. **C**. and **D**.: Comparison of real and column random sequences. The color scale for each graph is shown on the top of each panel. Here positive Z-score indicates enrichment of disorder score in naturally occurring sequences while negative Z-score indicates the reverse. Organisms are arranged according to their mid-point rooted species tree retrieved from NCBI taxonomic database with the help of their species taxonomic identifier. NCBI, the national center for biotechnology information.

Since most of the disorder prediction algorithms used in this study are trained on native protein datasets, their accuracy on randomly generated sequences (such as ours) is questionable. To test this, we specifically considered one method, IUPred (updated version IUPred2A [25]), which has never been trained on any specific dataset, and calculated Z-scores solely based on the prediction of this method. We obtained similar results (Figure S19) to those based on the consensus approaches (Figure 3, Figure S6), implying that our results do not depend on whether the algorithms were trained on natural protein datasets or not.

Considering both of these approaches (consensus approach 1 and consensus approach 2), we can conclude the following trends: 1) along the position eukaryotic proteins are more disordered than random sequences; 2) this trend is stronger near the terminal regions (specifically near the N-terminal region) rather than at the center of eukaryotic proteins; and 3) in prokaryotes this trend is not present or is weakly present near the terminal regions. In the next few sections, we explored the possible causes and consequences of these trends.

### Bias in disorder prediction at the terminal regions cannot explain the trends

**Supplementary figure S20:**
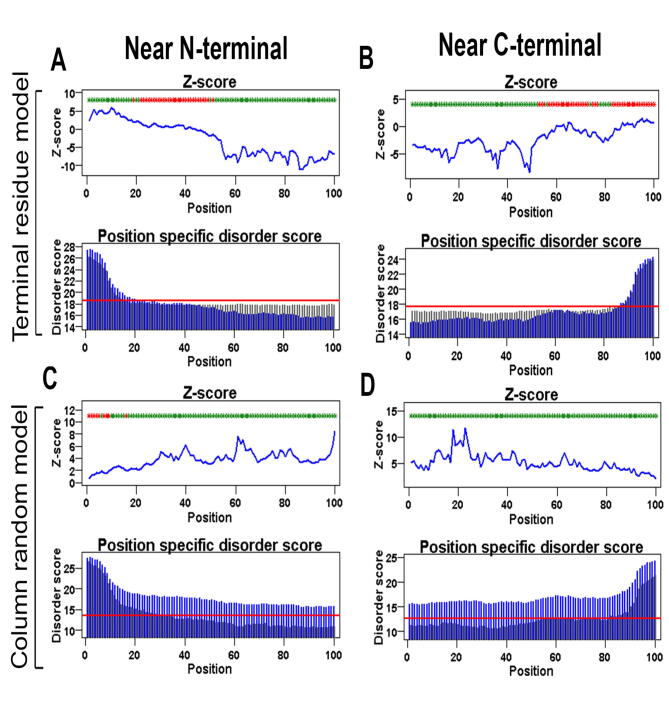
**Z-score profile for position-specific disorder scores of *A. oryzae* after removing the first and last 50 residues**. For this test we removed the terminal regions (up to the first and last 50 amino acid positions) from the extant protein sequences of *A. oryzae* and generated random models (column random and terminal residue random models) from this terminal regions removed real protein dataset. We freshly predicted disordered residues in these sequences (both real and random) following consensus approach 1 and analyzed position-wise Z-score profile as we did for the full-length sequences (see main text). This figure shows the extent of protein disorder between real and random protein models (generated for this test) up to the first 100 and last 100 positions. **A**. and **B**.: Comparison of real and terminal residue conserved random sequences. **C**. and **D**.: Comparison of real and column random sequences. In each panel, vertical blue lines stand for the average position-specific disorder scores of real proteins and gray lines that for random proteins. At each position, the statistical difference between these two measurements is accessed through Z-score (see main text) and is shown with blue line. Corresponding *P* value at each position is shown either with a green (*P* < 0.05) or red dot (*P* ≥ 0.05) above the Z-score. Here positive Z-score indicates enrichment of protein disorder in naturally occurring sequences while negative Z-score indicates the reverse.

**Supplementary figure S21:**
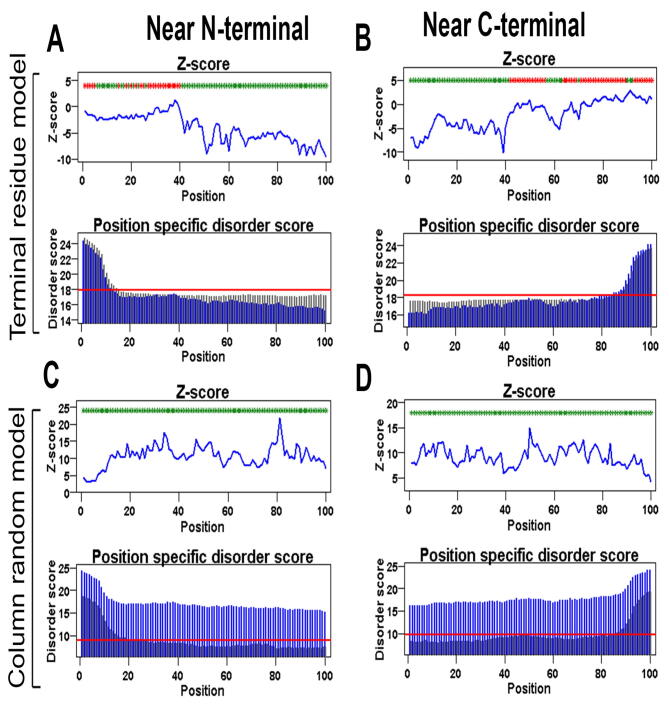
Z-score profile for position-specific disorder scores of *C. elegans* after removing the first and last 50 residues. For this test we removed the terminal regions (up to the first and last 50 amino acid positions) from the extant protein sequences of *C. elegans* and generated random models (column random and terminal residue random models) from this terminal regions removed real protein dataset. We freshly predicted disordered residues in these sequences (both real and random) following consensus approach 1 and analyzed position-wise Z-score profile as we did for the full-length sequences (see main text). This figure shows the extent of protein disorder between real and random protein models (generated for this test) up to the first 100 and last 100 positions. **A**. and **B**.: Comparison of real and terminal residue conserved random sequences. **C**. and **D**.: Comparison of real and column random sequences. In each panel, vertical blue lines stand for the average position-specific disorder scores of real proteins and gray lines that for random proteins. At each position, the statistical difference between these two measurements is accessed through Z-score (see main text) and is shown with blue line. Corresponding *P* value at each position is shown either with a green (*P* < 0.05) or red dot (*P* ≥ 0.05) above the Z-score. Here positive Z-score indicates enrichment of protein disorder in naturally occurring sequences while negative Z-score indicates the reverse.

**Supplementary figure S22:**
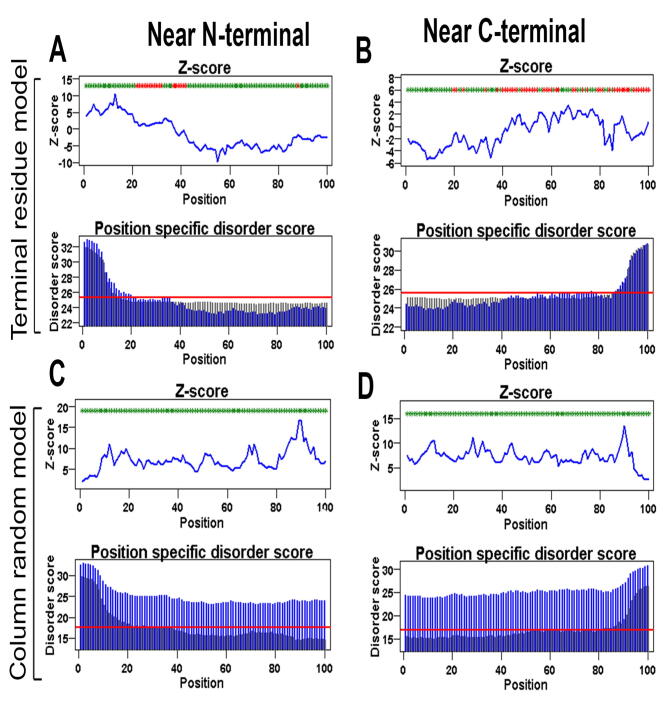
**Z-score profile for position-specific disorder scores of *D. melanogaster* after removing the first and last 50 residues**. For this test we removed the terminal regions (up to the first and last 50 amino acid positions) from the extant protein sequences of *D. melanogaster* and generated random models (column random and terminal residue random models) from this terminal regions removed real protein dataset. We freshly predicted disordered residues in these sequences (both real and random) following consensus approach 1 and analyzed position-wise Z-score profile as we did for the full-length sequences (see main text). This figure shows the extent of protein disorder between real and random protein models (generated for this test) up to the first 100 and last 100 positions. **A**. and **B**.: Comparison of real and terminal residue conserved random sequences. **C**. and **D**.: Comparison of real and column random sequences. In each panel, vertical blue lines stand for the average position-specific disorder scores of real proteins and gray lines that for random proteins. At each position, the statistical difference between these two measurements is accessed through Z-score (see main text) and is shown with blue line. Corresponding *P* value at each position is shown either with a green (*P* < 0.05) or red dot (*P* ≥ 0.05) above the Z-score. Here positive Z-score indicates enrichment of protein disorder in naturally occurring sequences while negative Z-score indicates the reverse.

**Supplementary figure S23:**
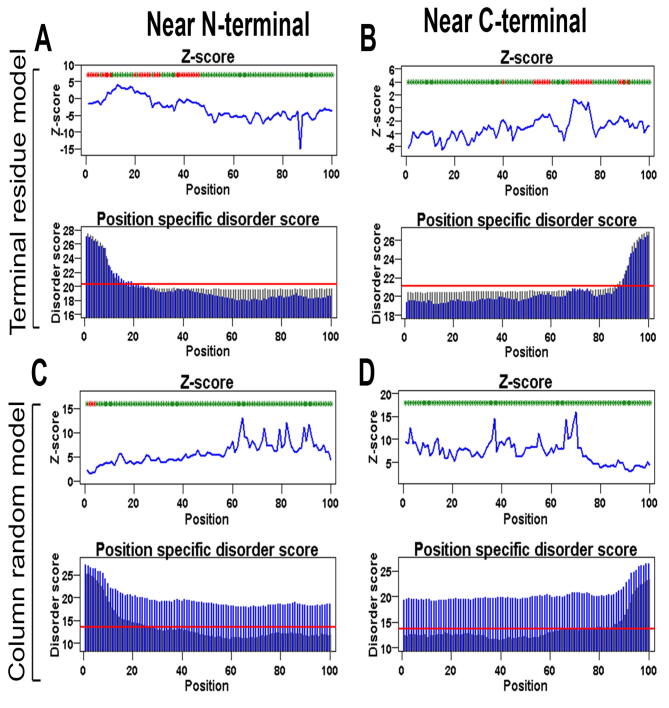
**Z-score profile for position-specific disorder scores of *H. sapiens* after removing the first and last 50 residues**. For this test we removed the terminal regions (up to the first and last 50 amino acid positions) from the extant protein sequences of *H. sapiens* and generated random models (column random and terminal residue random models) from this terminal regions removed real protein dataset. We freshly predicted disordered residues in these sequences (both real and random) following consensus approach 1 and analyzed position-wise Z-score profile as we did for the full-length sequences (see main text). This figure shows the extent of protein disorder between real and random protein models (generated for this test) up to the first 100 and last 100 positions. **A**. and **B**.: Comparison of real and terminal residue conserved random sequences. **C**. and **D**.: Comparison of real and column random sequences. In each panel, vertical blue lines stand for the average position-specific disorder scores of real proteins and gray lines that for random proteins. At each position, the statistical difference between these two measurements is accessed through Z-score (see main text) and is shown with blue line. Corresponding *P* value at each position is shown either with a green (*P* < 0.05) or red dot (*P* ≥ 0.05) above the Z-score. Here positive Z-score indicates enrichment of protein disorder in naturally occurring sequences while negative Z-score indicates the reverse.

**Supplementary figure S24:**
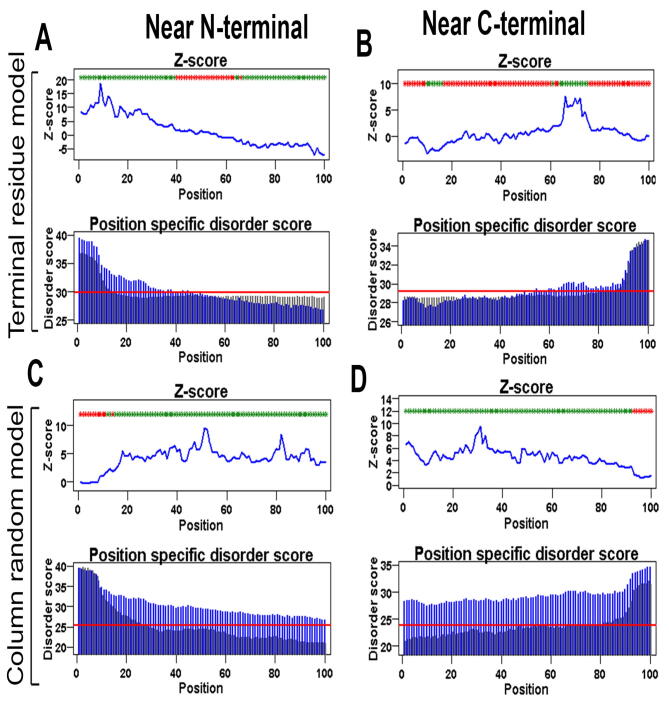
**Z-score profile for position-specific disorder scores of *N. crassa* after removing the first and last 50 residues**. For this test we removed the terminal regions (up to the first and last 50 amino acid positions) from the extant protein sequences of *N. crassa* and generated random models (column random and terminal residue random models) from this terminal regions removed real protein dataset. We freshly predicted disordered residues in these sequences (both real and random) following consensus approach 1 and analyzed position-wise Z-score profile as we did for the full-length sequences (see main text). This figure shows the extent of protein disorder between real and random protein models (generated for this test) up to the first 100 and last 100 positions. **A**. and **B**.: Comparison of real and terminal residue conserved random sequences. **C**. and **D**.: Comparison of real and column random sequences. In each panel, vertical blue lines stand for the average position-specific disorder scores of real proteins and gray lines that for random proteins. At each position, the statistical difference between these two measurements is accessed through Z-score (see main text) and is shown with blue line. Corresponding *P* value at each position is shown either with a green (*P* < 0.05) or red dot (*P* ≥ 0.05) above the Z-score. Here positive Z-score indicates enrichment of protein disorder in naturally occurring sequences while negative Z-score indicates the reverse.

**Supplementary figure S25:**
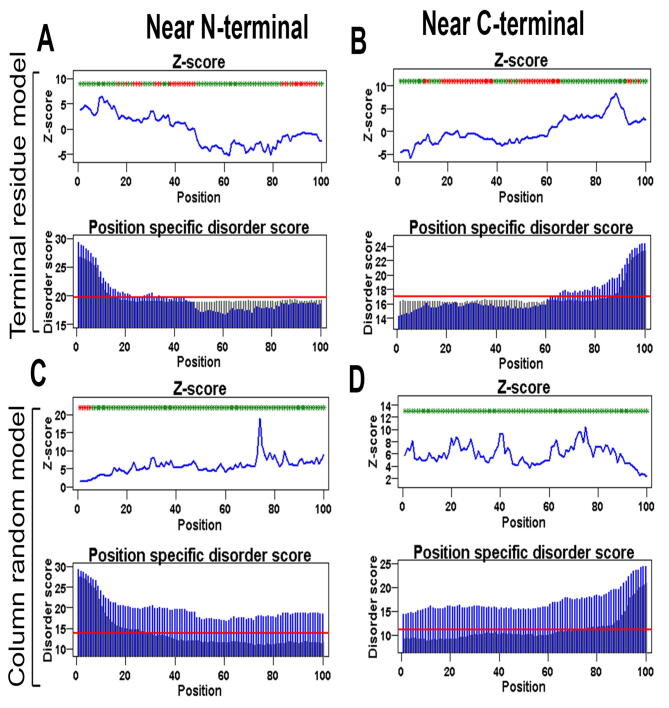
**Z-score profile for position-specific disorder scores of *S. cerevisiae* after removing the first and last 50 residues**. For this test we removed the terminal regions (up to the first and last 50 amino acid positions) from the extant protein sequences of *S. cerevisiae* and generated random models (column random and terminal residue random models) from this terminal regions removed real protein dataset. We freshly predicted disordered residues in these sequences (both real and random) following consensus approach 1 and analyzed position-wise Z-score profile as we did for the full-length sequences (see main text). This figure shows the extent of protein disorder between real and random protein models (generated for this test) up to the first 100 and last 100 positions. **A**. and **B**.: Comparison of real and terminal residue conserved random sequences. **C**. and **D**.: Comparison of real and column random sequences. In each panel, vertical blue lines stand for the average position-specific disorder scores of real proteins and gray lines that for random proteins. At each position, the statistical difference between these two measurements is accessed through Z-score (see main text) and is shown with blue line. Corresponding *P* value at each position is shown either with a green (*P* < 0.05) or red dot (*P* ≥ 0.05) above the Z-score. Here positive Z-score indicates enrichment of protein disorder in naturally occurring sequences while negative Z-score indicates the reverse.

We noticed that almost all disorder prediction algorithms predict very high disorder near the protein ends. Therefore, one probability may be that the ends of the proteins (irrespective of whether from protein terminal regions or not), in general, show the trends due to biases in disorder prediction. To check this possibility, we removed the terminal regions (up to the first 50 and last 50 residues where we found high positive Z-scores) from the native protein sequences of six eukaryotic organisms showing the trend. We generated random protein models (both terminal residue conserved random sequences and column-wise random sequences) from these truncated sequences, predicted disordered residues freshly in these (real and random) sequences, and compared their disorder scores following Z-score approach as described above. Considering both types of random models (column-wise random and terminal residue conserved random models), we found generally similar results corresponding to the analogous (50th–150th) positions in the full-length sequences (Figures S20–S25). For instance, in our main analysis when we compared the disorder scores of full-length native sequences in reference to the terminal residue conserved random model, we found weak positive Z-scores (compared to the end positions) starting from the 50th position of both terminal regions. Considering terminal residue conserved random model, in this analysis (with truncated sequences), we did not find any significant trend (high positive Z-score) near the C-terminal regions of most of our test eukaryotic organisms (panel B in Figures S20–S25). No positive trend (Z-score) is noticed near the N-terminal regions of *H. sapiens* (Figure S23A) and *C. elegans* (Figure S21A) proteins either. However, significant trends were noticed near the N-terminal regions of other eukaryotes like *S. cerevisiae* (Figure S25A), *N. crassa* (Figure S24A), and *D. melanogaster* (Figure S22A). This is generally weaker than the N-terminal regions of their full-length sequences and similar to the level near the 50th position of the corresponding full-length sequences. Considering column-wise random sequences, in our full-length protein datasets, we found more or less similar Z-scores throughout the length of the protein. Using truncated sequences we found similar but generally weaker scores near the truncated terminal regions when we considered column-wise random sequences as reference (panels C and D in Figures S20–S25). Overall, the results are similar to those obtained at the analogous positions of full-length protein sequences. Thus, the trends that we found near the terminal regions of eukaryotic proteins cannot be reproduced in any other position generating artificial protein ends.

### Selection for high GC content at the nucleotide level cannot explain the trends

**Supplementary figure S26:**
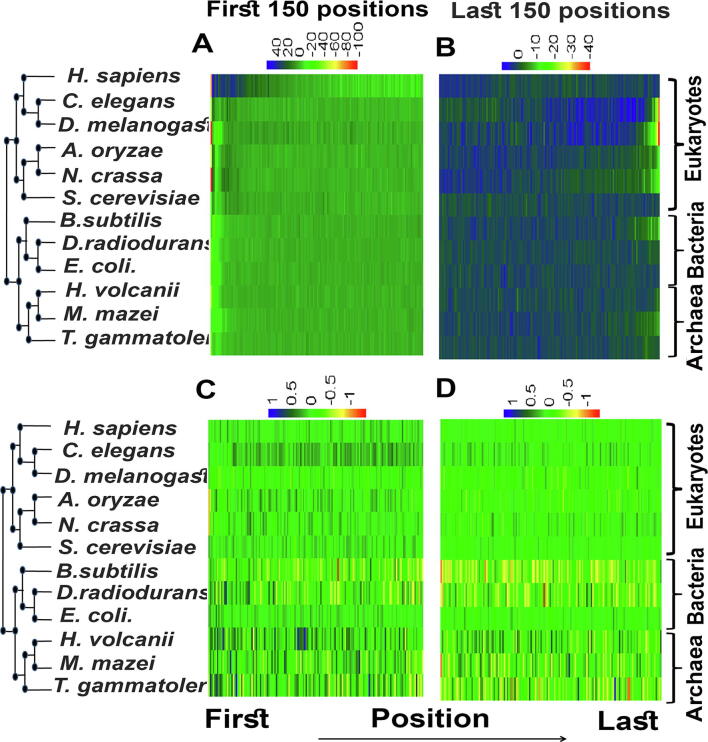
**Z-score profile for GC content for the 12 species considered in this study**. This figure shows the Z-score profile calculated comparing GC content of real and randomly generated nucleotide sequences for the first 150 and last 150 positions of 12 organisms considered in this study. In panels **A** and **B**, Z-scores were calculated comparing GC content of real and terminal residue conserved random sequences of each species and in panels **C** and **D**, Z-scores were calculated comparing GC content of real and column random sequences of each species. The color scale for each graph is shown on the top of each panel. Here positive Z-score indicates enrichment of GC content in naturally occurring sequences while negative Z-score indicates the reverse. Organisms are arranged according to their mid-point rooted species tree retrieved from NCBI taxonomic database with the help of their species taxonomic identifier. NCBI, the national center for biotechnology information.

The results presented so far reveal a general (proteome-wise disorder content) and regional (near the terminal regions) enrichment of disordered residues in eukaryotic proteins compared to their corresponding random sequences. Protein disorder content was suggested to depend on several factors, among which genomic GC content [16], [17], [26] is considered the most significant. Further, significant correlations (Table 1) were found between protein disorder content and genomic GC content in each species, suggesting that the observed trends may be due to the selection for high or low GC content at the nucleotide level. In the aforementioned section, we showed that the trend that we found considering the disorder content of full-length proteins is valid over the entire GC range of their coding sequences (Figures S4 and S5). Now, in this section, we checked the second possibility whether the enrichment of protein disorder near the N- and C-terminal regions of eukaryotic proteins is the results of selection for high GC content in their coding sequences. To explore this possibility, we compared the GC content of real protein-coding sequences of each test species with that of randomly generated nucleotide sequences position-wise. To this end, we generated random nucleotide sequences analogous to terminal residue conserved and column-wise random protein models and calculated Z-scores which signify the deviation in GC content between real and random sequences. The GC profiles near the protein terminals with respect to terminal residue conserved and column-wise random nucleotide models are shown in Figure S26. This result suggests that in most of the species there is either no or weak evidence of preference (*P* > 0.05) for high or low GC near the protein terminal regions. If GC content has any impact, we would expect to see a concomitant trend to what we observed for disordered residues in all the tested species. To further probe the possibility of any hidden link between selection at the nucleotide and protein levels, we correlated Z-scores for disordered residues with corresponding Z-scores for GC content. We did not find any significant correlation between the two measures in any of the tested species. These results suggest that the observed trends of high disorder near the terminal regions of eukaryotic proteins are independent of selection for higher GC content at the nucleotide level.

**Table 1:** Correlation between GC content and protein disorder content

Lineage	Species name	Sample size (N)	Correlation between GC content and % of Dis_all residues	Correlation between GC content and % of Dis_30 residues
Mammal	*Homo sapiens*	16,384	*ρ* = 0.235; *P* < 1 × 10^−6^	*ρ* = 0.189; *P* < 1 × 10^−6^
Insect	*Drosophila melanogaster*	24,799	*ρ* = 0.300; *P* < 1 × 10^−6^	*ρ* = 0.279; *P* < 1 × 10^−6^
Worm	*Caenorhabditis elegans*	21,187	*ρ* = 0.387; *P* < 1 × 10^−6^	*ρ* = 0.312; *P* < 1 × 10^−6^
Fungi	*Saccharomyces cerevisiae*	4772	*ρ* = 0.114; *P* < 1 × 10^−6^	*ρ* = 0.049; *P* = 2.05 × 10^−4^
Fungi	*Aspergillus oryzae*	9830	*ρ* = 0.228; *P* < 1 × 10^−6^	*ρ* = 0.194; *P* < 1 × 10^−6^
Fungi	*Neurospora crassa*	8899	*ρ* = 0.082; *P* < 1 × 10^−6^	*ρ* = 0.068; *P* < 1 × 10^−6^
Bacteria	*Bacillus subtilis*	2588	*ρ* = 0.075; *P* = 2 × 10^−6^	*ρ* = -0.028; *P* = 7.2 × 10^−2^
Bacteria	*Escherichia coli*	2838	*ρ* = 0.101; *P* < 1 × 10^−6^	*ρ* = 0.061; *P =* 1.11 × 10^−4^
Bacteria	*Deinococcus radiodurans*	2080	*ρ* = 0.150; *P* < 1 × 10^−6^	*ρ* = 0.066; *P* = 2.13 × 10^−4^
Archaea	*Methanosarcina mazei*	2063	*ρ* = 0.135; *P* < 1 × 10^−6^	*ρ* = 0.071; *P* = 4.50 × 10^−5^
Archaea	*Haloferax volcanii*	2410	*ρ* = 0.072; *P* < 8 × 10^−6^	*ρ* = 0.086; *P* < 1 × 10^−6^

*Note*: This table shows the correlation between disorder content of naturally occurring proteins in each species with the GC content of their coding sequences. Disorder content of a protein was calculated considering 1) all the predicted disordered residues (denoted as percentage of all disordered residues, % of Dis_all residues) and 2) disordered residues only in long disordered regions (denoted as percentage of 30 or more consecutive disordered residues, % of Dis_30 residues). In each protein, disordered residues were predicted by consensus-based approach 1 (see main text). Following non-parametric distribution of protein disorder content, here we calculated Spearman’s Rank correlation coefficient ρ, where *P* values stand for significance level. N stands for sample size, *i.e.*, the number of proteins and their coding sequences considered in each species for this test.

### Splice junctions cannot explain the trends

**Supplementary figure S27:**
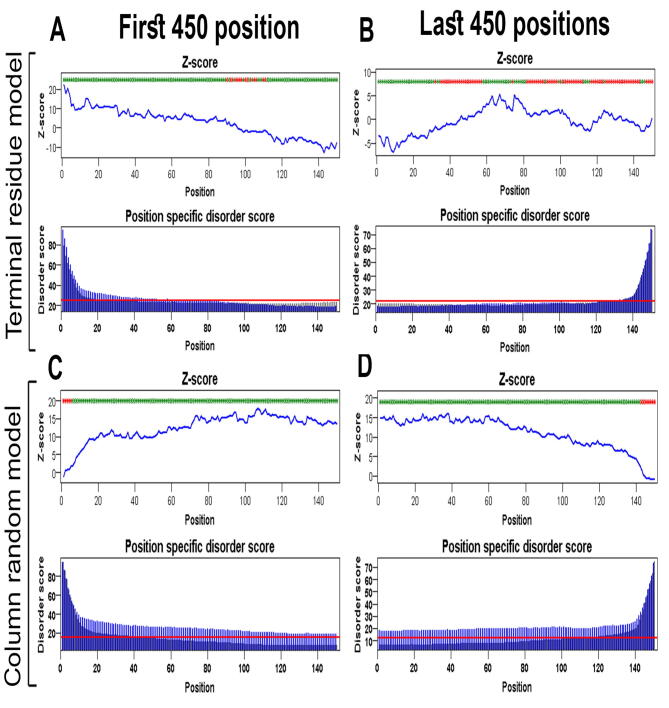
**Z-score profile for the position-specific disorder score of *A. oryzae* considering proteins without any splice junction in the coding sequences up to the first and last 100 amino acids positions**. This figure shows the extent of protein disorder in the proteins without any splice junction in the coding sequences up to the first and last 100 amino acids positions and the corresponding random sequences specifically generated from these sequences. Disordered residues in these real and random protein datasets were predicted by consensus approach 1 (see main text for details). In each panel, vertical blue lines stand for the average position-specific disorder scores of real proteins and gray lines that for random proteins. At each position, the statistical difference between these two measurements is accessed through Z-score (see main text) and is shown with blue line. In panels **A** and **B**, Z-scores were calculated comparing position-specific disorder scores of real and terminal residue conserved random sequences of each species and in panels **C** and **D**, Z-scores were calculated comparing position-specific disorder scores of real and column random sequences of each species. Here positive Z-score indicates enrichment of protein disorder in naturally occurring sequences while negative Z-score indicates the reverse. Corresponding *P* value at each position is shown either with a green (*P* < 0.05) or red dot (*P* ≥ 0.05) above the Z-score.

**Supplementary figure S28:**
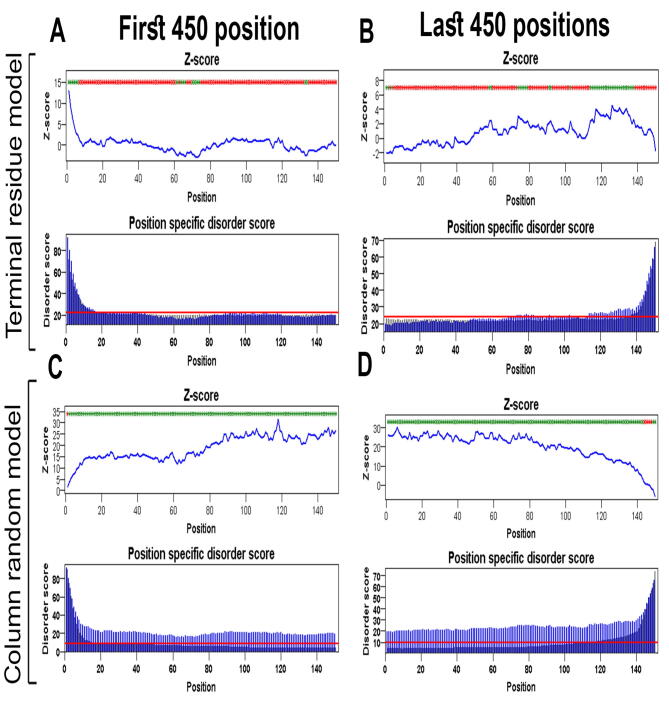
**Z-score profile for the position-specific disorder score of *C. elegans* considering proteins without any splice junction in the coding sequences up to the first and last 100 amino acids positions**. This figure shows the extent of protein disorder in the proteins without any splice junction in the coding sequences up to the first and last 100 amino acids positions and the corresponding random sequences specifically generated from these sequences. Disordered residues in these real and random protein datasets were predicted by consensus approach 1 (see main text for details). In each panel, vertical blue lines stand for the average position-specific disorder scores of real proteins and gray lines that for random proteins. At each position, the statistical difference between these two measurements is accessed through Z-score (see main text) and is shown with blue line. In panels **A** and **B**, Z-scores were calculated comparing position-specific disorder scores of real and terminal residue conserved random sequences of each species and in panels **C** and **D**, Z-scores were calculated comparing position-specific disorder scores of real and column random sequences of each species. Here positive Z-score indicates enrichment of protein disorder in naturally occurring sequences while negative Z-score indicates the reverse. Corresponding *P* value at each position is shown either with a green (*P* < 0.05) or red dot (*P* ≥ 0.05) above the Z-score.

**Supplementary figure S29:**
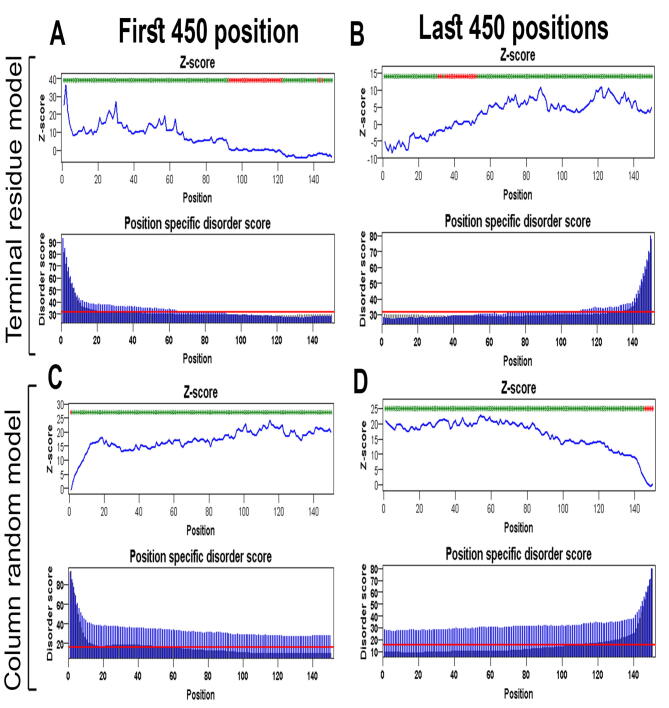
**Z-score profile for the position-specific disorder score of *D. melanogaster* considering proteins without any splice junction in the coding sequences up to the first and last 100 amino acids positions**. This figure shows the extent of protein disorder in the proteins without any splice junction in the coding sequences up to the first and last 100 amino acids positions and the corresponding random sequences specifically generated from these sequences. Disordered residues in these real and random protein datasets were predicted by consensus approach 1 (see main text for details). In each panel, vertical blue lines stand for the average position-specific disorder scores of real proteins and gray lines that for random proteins. At each position, the statistical difference between these two measurements is accessed through Z-score (see main text) and is shown with blue line. In panels **A** and **B**, Z-scores were calculated comparing position-specific disorder scores of real and terminal residue conserved random sequences of each species and in panels **C** and **D**, Z-scores were calculated comparing position-specific disorder scores of real and column random sequences of each species. Here positive Z-score indicates enrichment of protein disorder in naturally occurring sequences while negative Z-score indicates the reverse. Corresponding *P* value at each position is shown either with a green (*P* < 0.05) or red dot (*P* ≥ 0.05) above the Z-score.

**Supplementary figure S30:**
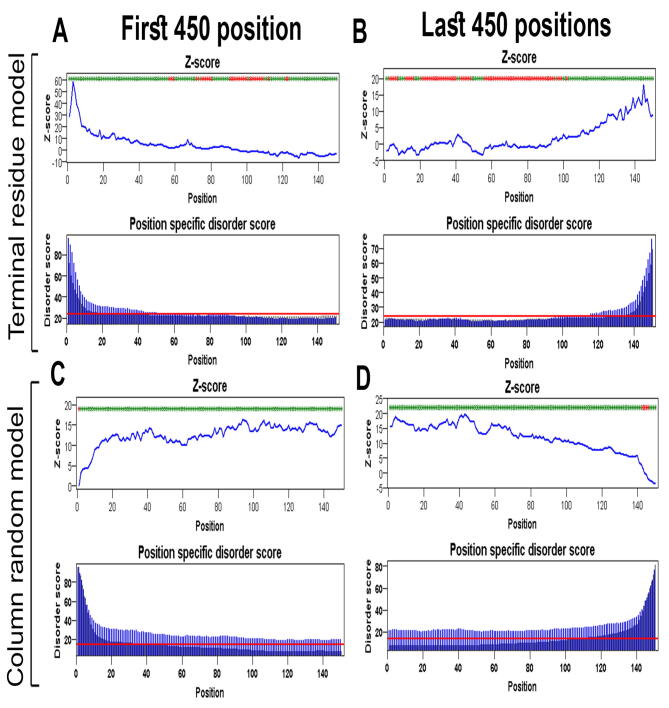
**Z-score profile for the position-specific disorder score of *H. sapiens* considering proteins without any splice junction in the coding sequences up to the first and last 100 amino acids positions**. This figure shows the extent of protein disorder in the proteins without any splice junction in the coding sequences up to the first and last 100 amino acids positions and the corresponding random sequences specifically generated from these sequences. Disordered residues in these real and random protein datasets were predicted by consensus approach 1 (see main text for details). In each panel, vertical blue lines stand for the average position-specific disorder scores of real proteins and gray lines that for random proteins. At each position, the statistical difference between these two measurements is accessed through Z-score (see main text) and is shown with blue line. In panels **A** and **B**, Z-scores were calculated comparing position-specific disorder scores of real and terminal residue conserved random sequences of each species and in panels **C** and **D**, Z-scores were calculated comparing position-specific disorder scores of real and column random sequences of each species. Here positive Z-score indicates enrichment of protein disorder in naturally occurring sequences while negative Z-score indicates the reverse. Corresponding *P* value at each position is shown either with a green (*P* < 0.05) or red dot (*P* ≥ 0.05) above the Z-score.

**Supplementary figure S31:**
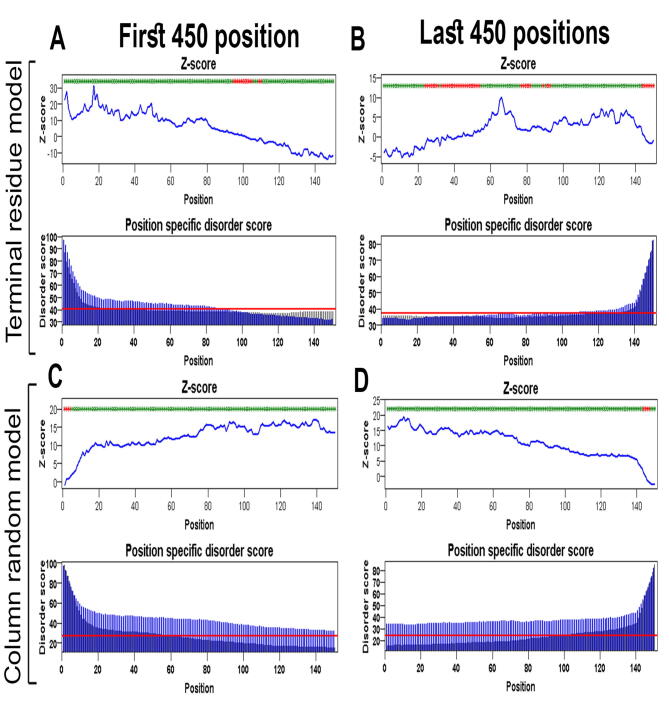
**Z-score profile for the position-specific disorder score of *N. crassa* considering proteins without any splice junction in the coding sequences up to the first and last 100 amino acids positions**. This figure shows the extent of protein disorder in the proteins without any splice junction in the coding sequences up to the first and last 100 amino acids positions and the corresponding random sequences specifically generated from these sequences. Disordered residues in these real and random protein datasets were predicted by consensus approach 1 (see main text for details). In each panel, vertical blue lines stand for the average position-specific disorder scores of real proteins and gray lines that for random proteins. At each position, the statistical difference between these two measurements is accessed through Z-score (see main text) and is shown with blue line. In panels **A** and **B**, Z-scores were calculated comparing position-specific disorder scores of real and terminal residue conserved random sequences of each species and in panels **C** and **D**, Z-scores were calculated comparing position-specific disorder scores of real and column random sequences of each species. Here positive Z-score indicates enrichment of protein disorder in naturally occurring sequences while negative Z-score indicates the reverse. Corresponding P value at each position is shown either with a green (*P* < 0.05) or red dot (*P* ≥ 0.05) above the Z-score.

**Supplementary figure S32:**
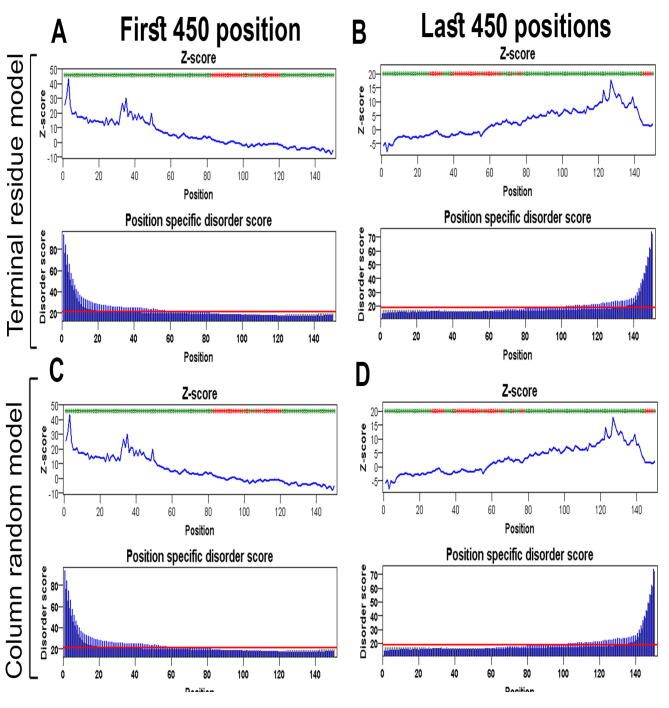
**Z-score profile for the position-specific disorder score of *S. cerevisiae* considering proteins without any splice junction in the coding sequences up to the first and last 100 amino acids positions**. This figure shows the extent of protein disorder in the proteins without any splice junction in the coding sequences up to the first and last 100 amino acids positions and the corresponding random sequences specifically generated from these sequences. Disordered residues in these real and random protein datasets were predicted by consensus approach 1 (see main text for details). In each panel, vertical blue lines stand for the average position-specific disorder scores of real proteins and gray lines that for random proteins. At each position, the statistical difference between these two measurements is accessed through Z-score (see main text) and is shown with blue line. In panels **A** and **B**, Z-scores were calculated comparing position-specific disorder scores of real and terminal residue conserved random sequences of each species and in panels **C** and **D**, Z-scores were calculated comparing position-specific disorder scores of real and column random sequences of each species. Here positive Z-score indicates enrichment of protein disorder in naturally occurring sequences while negative Z-score indicates the reverse. Corresponding *P* value at each position is shown either with a green (*P* < 0.05) or red dot (*P* ≥ 0.05) above the Z-score.

Previously it was shown that disordered residues are more prevalent near splice junctions of coding sequences [27]. Therefore, the trends we observed near the terminal regions of eukaryotic proteins may be caused by proteins having more splice junctions in their coding sequences near those regions. To check this possibility, we considered only proteins without any splice junction in their coding sequences up to the first 100 and last 100 positions (*i.e.,* those encoded by a single exon or those in which the first and last exons are more than 300 bp in length). We tested six eukaryotic organisms in which we found a higher fraction of protein disordered residues in real sequences compared to the random models. When compared position-specific disorder scores of these proteins with their corresponding terminal residue conserved and column-wise random sequences (specifically generated from these sequences) in most of the eukaryotes, we found similar trends to those found considering all proteins (Figures S27–S32), which suggests that splice junctions don’t have any major effect on our observed trends.

### High solvent accessibility near the terminal regions of proteins cannot explain the disorder trends

**Supplementary figure S33:**
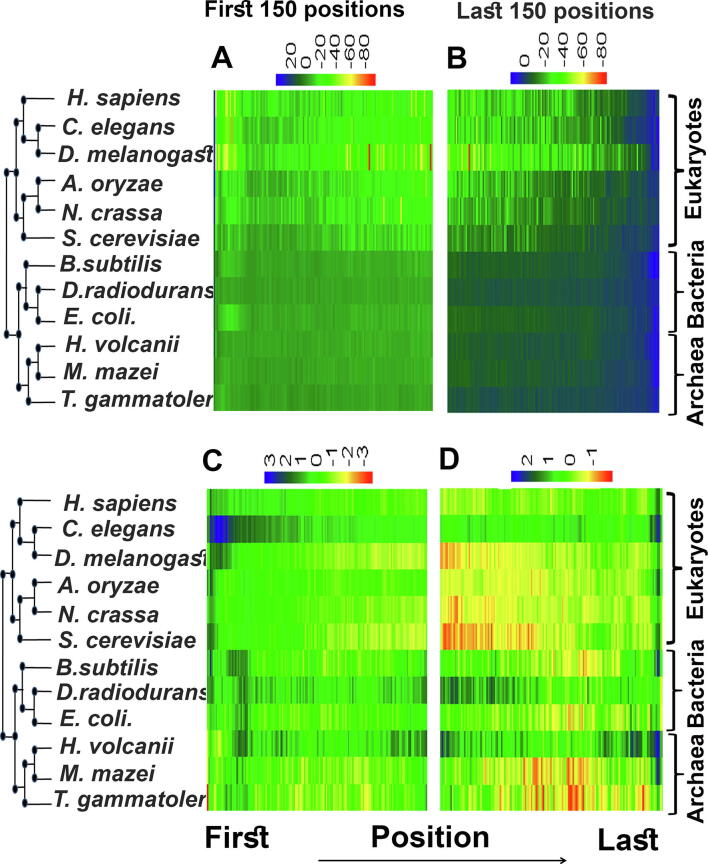
**Z-score profile for the predicted solvent accessibility for the 12 species considered in this study**. This figure shows the Z-score profile for the predicted solvent accessibility of real and randomly generated protein sequences for the first 150 and last 150 positions of 12 organisms considered in this study. In panels **A** and **B**, Z-scores were calculated comparing position-specific disorder scores of real and terminal residue conserved random sequences of each species and in panels **C** and **D**, Z-scores were calculated comparing position-specific disorder scores of real and column random sequences of each species. The color scale for each graph is shown on the top of each panel. Here positive Z-score indicates enrichment of predicted solvent accessibility in naturally occurring sequences while negative Z-score indicates the reverse. Organisms are arranged according to their mid-point rooted species tree retrieved from NCBI taxonomic database with the help of their species taxonomic identifier. NCBI, the national center for biotechnology information.

Previously, terminal regions of proteins were shown to be solvent-exposed [28]. This solvent-exposed nature of terminal regions was suggested to arise from excessive use of hydrophilic and polar residues [28] which are known to increase the propensity of a protein to be disordered [1], [2], [3]. Consequently, high protein disorder near the terminal regions of eukaryotic proteins may be a side effect of charged residues selected mainly for the solvent-exposed nature of these regions. To test this possibility, we calculated Z-score profiles for predicted solvent accessibility by using a similar approach to that used for predicted disordered residues. Using both random models (terminal residue conserved random model and column-wise random model), we did not find any strong evidence in any of our test organisms that real proteins show a preference for higher solvent accessibility near their terminal regions compared to random expectations (Figure S33). Moreover, we did not find any significant correlation between the Z-scores for predicted disordered regions and the Z-scores for predicted solvent accessibility, further suggesting that the trends we observed near the terminal regions of eukaryotic proteins are independent of their high solvent-exposed nature.

### Most human gene ontology functional categories show the reported disorder trends

**Figure 4:**
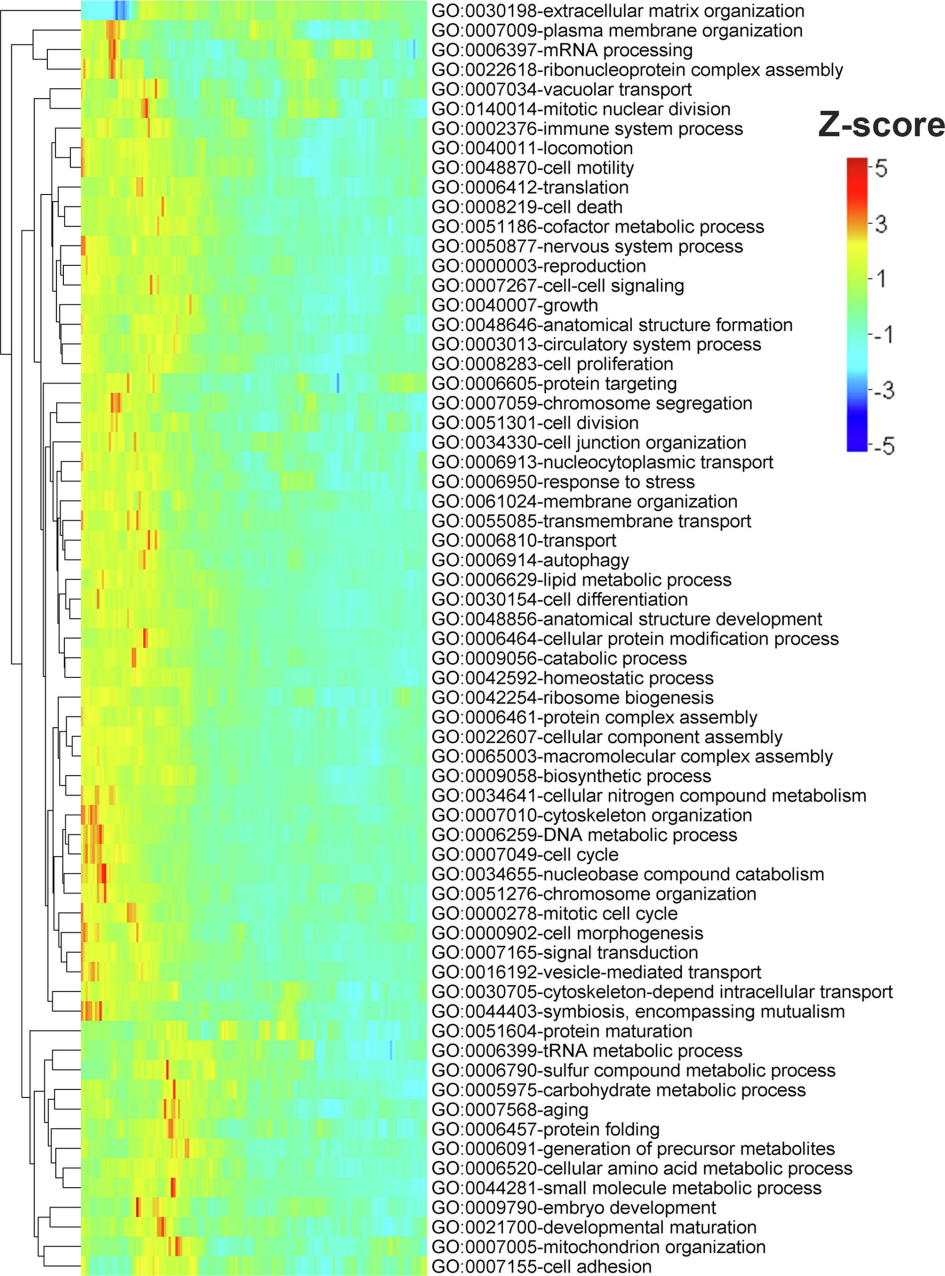
**Mean Z-score profile for the first 150 positions of human proteins under gene ontology (GO-slim) biological processcategoryin reference to terminal residue conserved random model** This heat map represents position-wise Z-score of predicted disordered residues (predicted by consensus approach 1) for the first 150 positions at the N-terminal regions of human proteins under each GO-slim biological process term. In each functional term, Z-scores for predicted disordered residues were calculated by considering proteins (more than 200 residues in length) under that term in reference to their corresponding terminal residue conserved random sequences. Here rows represent the positions along the protein sequence. Z-scores were coded in color scheme (color legend). Only the terms with more than 100 proteins are shown here.

**Supplementary figure S34:**
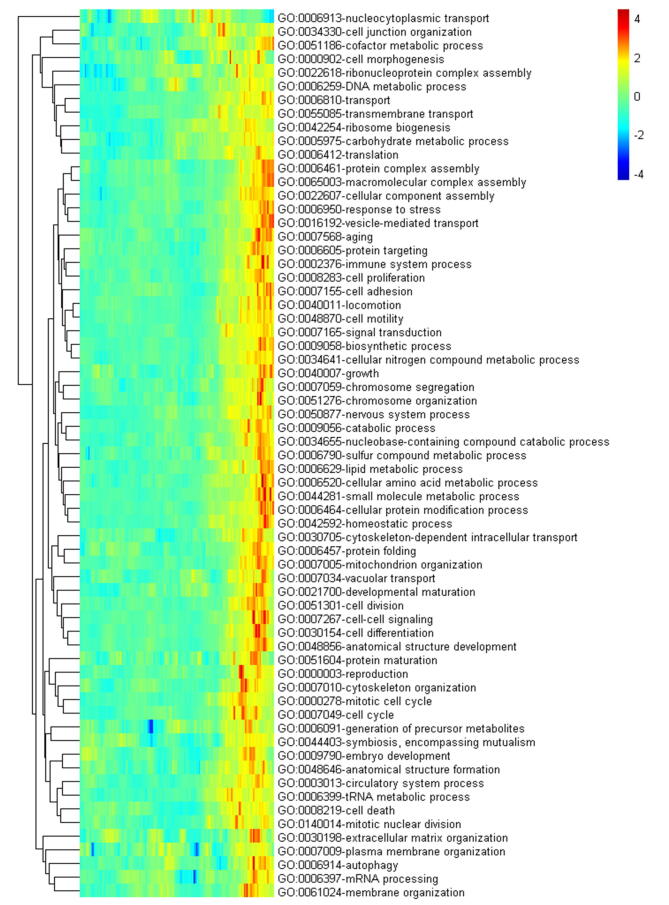
**Mean Z-score profile for the last 150 positions of *H. sapiens* proteins under GO slim biological process ontologies in reference to terminal residue conserved random**. This heat map represents position-wise Z-score of predicted disordered residues for the last 150 positions near the C-terminal region of human proteins under each GO slim biological process ontology. For each such GO-slim category, Z-scores for predicted disordered residues were calculated considering proteins (more than 200 residues in length) under that category in reference to their corresponding terminal residue conserved random sequences. Here rows represent the positions along the protein sequence. Z-scores were coded in color scheme (color legend). Only the ontologies with more than 100 proteins were shown. GO, gene ontology.

**Supplementary figure S35:**
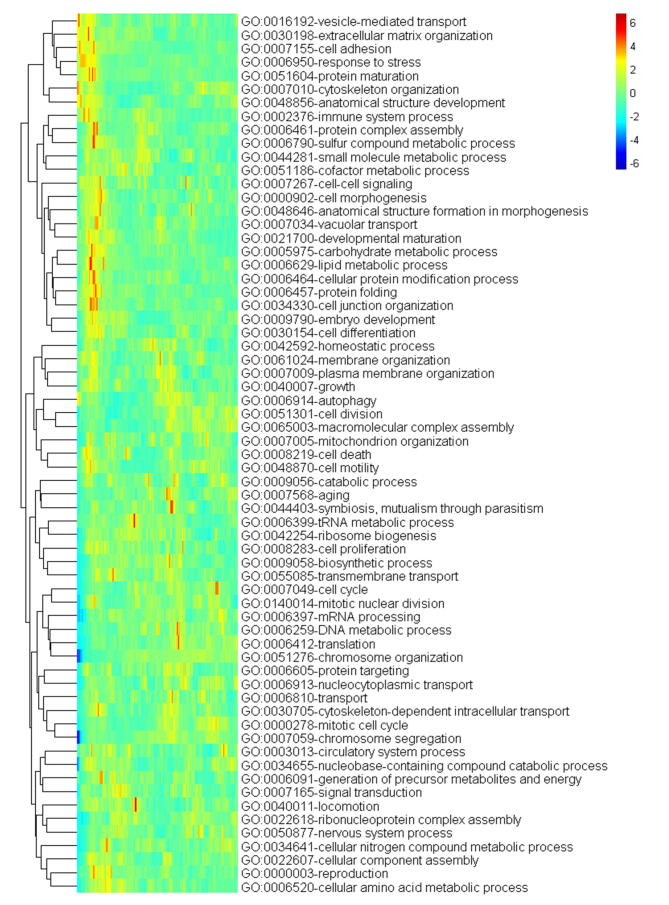
**Mean Z-score profile for the first 150 positions of *H. sapiens* proteins under GO slim biological process ontologies in reference to column random model**. This heat map represents position-wise Z-score of predicted disordered residues for the first 150 positions near the N-terminal region of human proteins under each GO slim biological process ontology. In each functional category, Z-scores for predicted disordered residues were calculated considering proteins (more than 200 residues in length) under that category in reference to their corresponding column random sequences. Here rows represent the positions along the protein sequence and columns represents Z-score coded in color scheme (color legend). Only the ontologies with more than 100 proteins were shown. GO, gene ontology.

**Supplementary figure S36:**
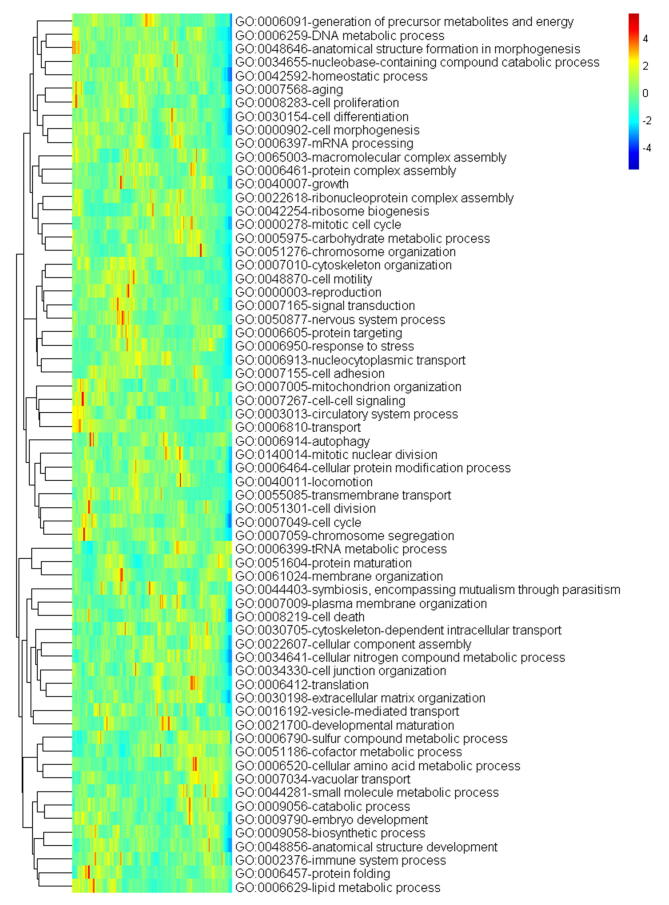
**Mean Z-score profile for the last 150 positions of *H. sapiens* proteins under GO slim biological process ontologies in reference to column random model**. This heat map represents position-wise Z-score of predicted disordered residues for the last 150 positions near the C-terminal region of *H. sapiens* proteins under each GO slim biological process ontology. In each functional category, Z-scores for predicted disordered residues were calculated considering proteins (more than 200 residues in length) under that category in reference to their corresponding column random sequences. Here rows represent the positions along the protein sequence and columns represents Z-score coded in color scheme (color legend). Only the ontologies with more than 100 proteins were shown. GO, gene ontology.

**Supplementary figure S37:**
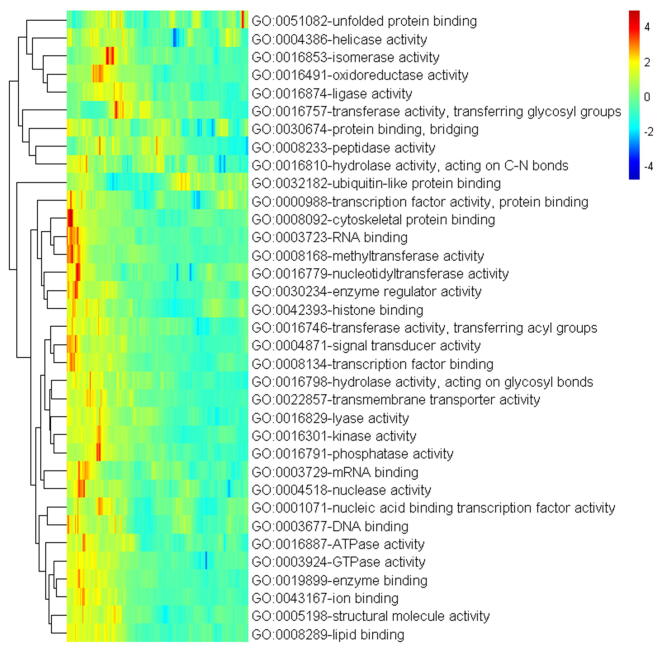
**Mean Z-score profile for the first 150 positions of *H. sapiens* proteins under GO slim molecular function ontologies in reference to terminal residue conserved random model**. This heat map represents position-wise Z-score of predicted disordered residues for the first 150 positions near the N-terminal region of *H. sapiens* proteins under each GO slim molecular function ontology. In each functional category, Z-scores for predicted disordered residues were calculated considering proteins (more than 200 residues in length) under that category in reference to their corresponding terminal residue conserved random sequences. Here rows represent the positions along the protein sequence and columns represents Z-score coded in color scheme (color legend). Only the ontologies with more than 100 proteins were shown. GO, gene ontology.

**Supplementary figure S38:**
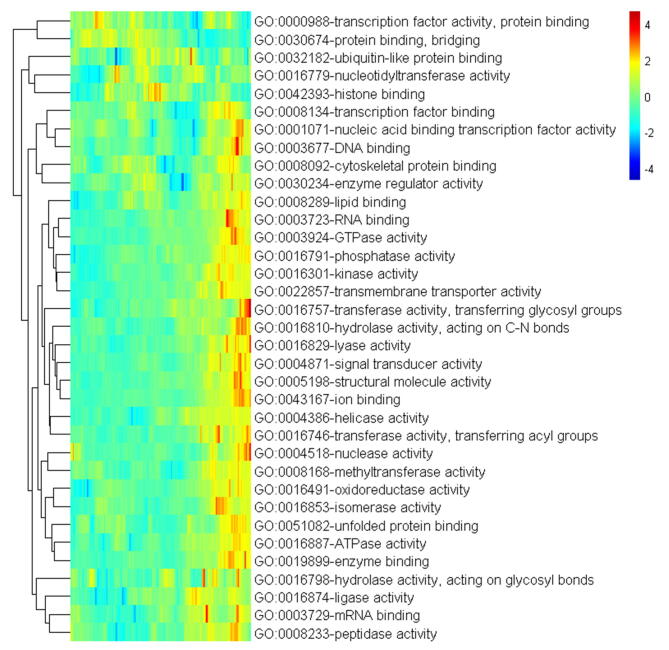
**Mean Z-score profile for the last 150 positions of *H. sapiens* proteins under GO slim molecular function ontologies in reference to terminal residue conserved random model**. This heat map represents position-wise Z-score of predicted disordered residues for the last 150 positions near the C-terminal region of H. sapiens proteins under each GO slim molecular function ontology. In each functional category, Z-scores for predicted disordered residues were calculated considering proteins (more than 200 residues in length) under that category in reference their corresponding terminal residue conserved random sequences. Here rows represent the positions along the protein sequence and columns represents Z-score coded in color scheme (color legend). Only the ontologies with more than 100 proteins were shown. GO, gene ontology.

**Supplementary figure S39:**
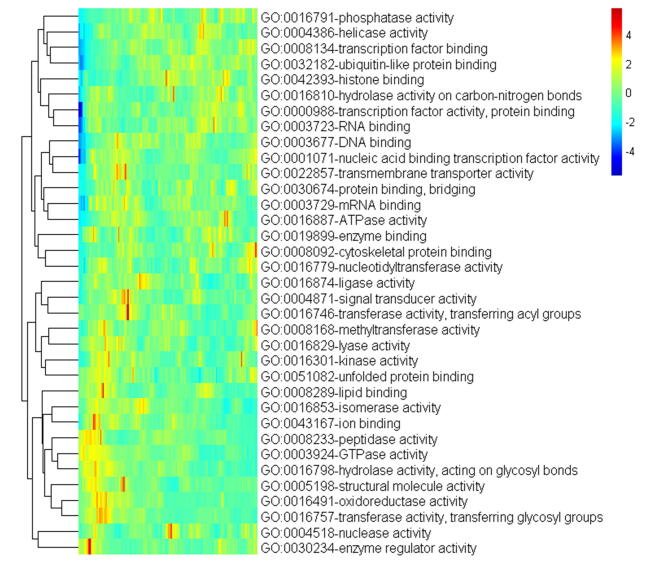
**Mean Z-score profile for the first 150 positions of *H. sapiens* proteins under GO slim molecular function ontologies in reference to column random model**. This heat map represents position-wise Z-score of predicted disordered residues for the first 150 positions near the N-terminal region of *H. sapiens* proteins under each GO slim molecular function ontology. In each functional category, Z-scores for predicted disordered residues were calculated considering proteins (more than 200 residues in length) under that category in reference to their corresponding column random sequences. Here rows represent the positions along the protein sequence and columns represents Z-score coded in color scheme (color legend). Only the ontologies with more than 100 proteins were shown. GO, gene ontology.

**Supplementary figure S40:**
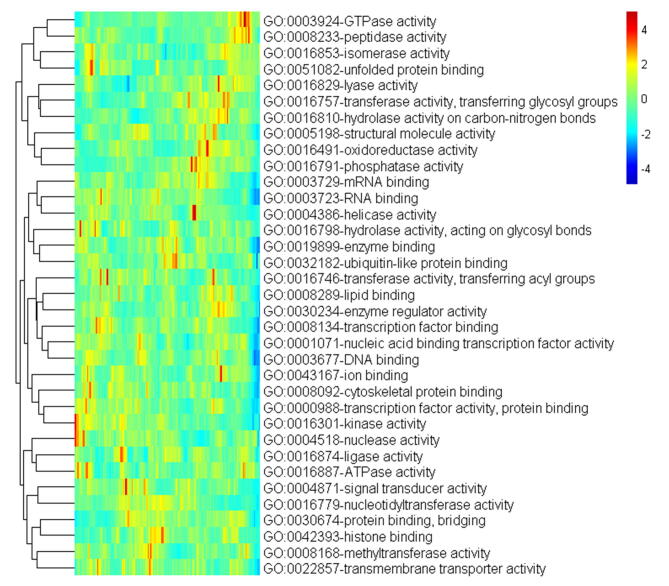
**Mean Z-score profile for the last 150 positions of *H. sapiens* proteins under GO slim molecular function ontologies in reference to column random model**. This heat map represents position-wise Z-score of predicted disordered residues for the last 150 positions near the C-terminal region of *H. sapiens* proteins under each GO slim molecular function ontology. In each functional category, Z-scores for predicted disordered residues were calculated considering proteins (more than 200 residues in length) under that category in reference to their corresponding column random sequences. Here rows represent the positions along the protein sequence and columns represents Z-score coded in color scheme (color legend). Only the ontologies with more than 100 proteins were shown. GO, gene ontology.

**Supplementary figure S41:**
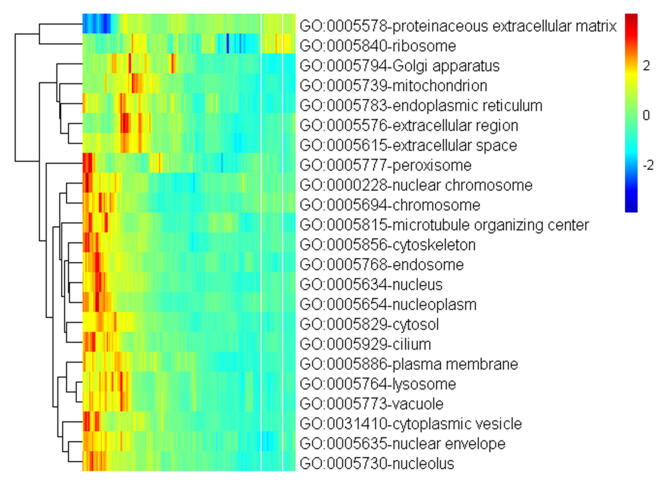
**Mean Z-score profile for the first 150 positions of *H. sapiens* proteins under their GO slim cellular component ontologies in reference to terminal residue conserved random model**. This heat map represents position-wise Z-score of predicted disordered residues for the first 150 positions near the N-terminal region of *H. sapiens* proteins under each GO slim cellular component ontology. In each functional category, Z-scores for predicted disordered residues were calculated considering proteins (more than 200 residues in length) under that category in reference to their corresponding terminal residue random sequences. Here rows represent the positions along the protein sequence and columns represents Z-score coded in color scheme (color legend). Only the ontologies with more than 100 proteins were shown. GO, gene ontology.

**Supplementary figure S42:**
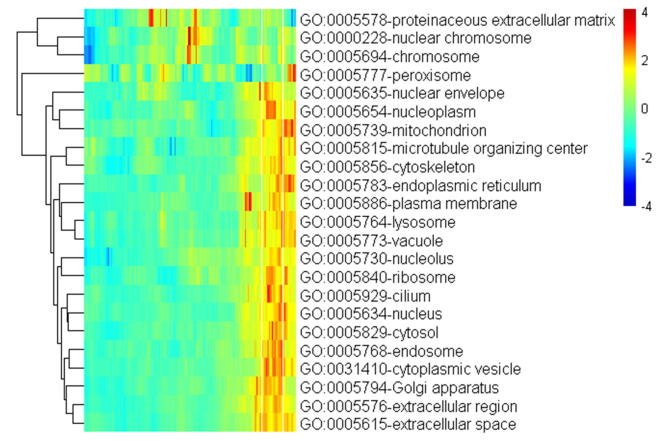
**Mean Z-score profile for the last 150 positions of *H. sapiens* proteins under GO slim cellular component ontologies in reference to terminal residue conserved random model**. This heat map represents position-wise Z-score of predicted disordered residues for the last 150 positions near the C-terminal region of *H. sapiens* proteins under each GO slim cellular component ontology. In each functional category, Z-scores for predicted disordered residues were calculated considering proteins (more than 200 residues in length) under that category in reference to their corresponding terminal residue conserved random sequences. Here rows represent the positions along the protein sequence and columns represents Z-score coded in color scheme (color legend). Only the ontologies with more than 100 proteins were shown. GO, gene ontology.

**Supplementary figure S43:**
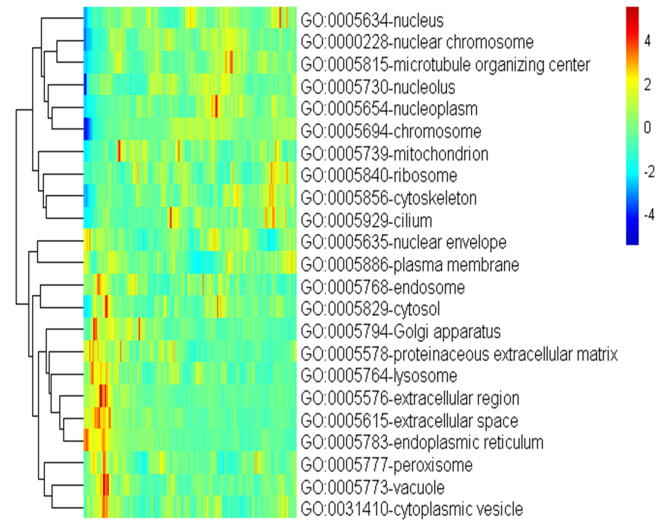
**Mean Z-score profile for the first 150 positions of *H. sapiens* proteins under GO slim cellular component ontologies in reference to column random model**. This heat map represents position-wise Z-score of predicted disordered residues for the first 150 positions near the N-terminal region of *H. sapiens* proteins under each GO slim cellular component ontology. In each functional category, Z-scores for predicted disordered residues were calculated considering proteins (more than 200 residues in length) under that category in reference to their corresponding column random sequences. Here rows represent the positions along the protein sequence and columns represents Z-score coded in color scheme (color legend). Only the ontologies with more than 100 proteins were shown. GO, gene ontology.

**Supplementary figure S44:**
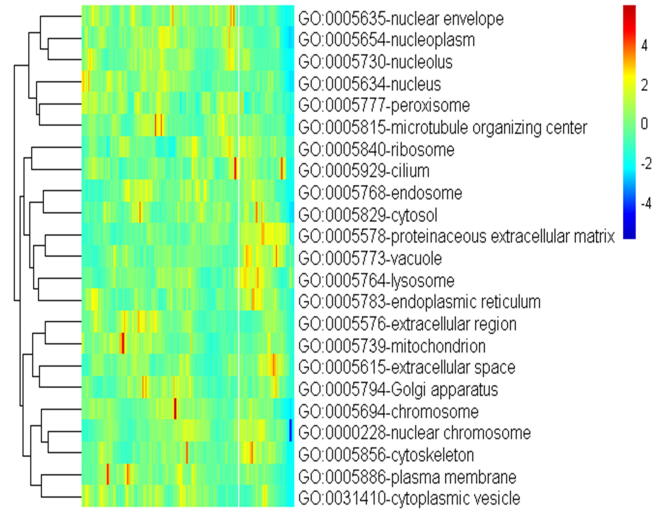
**Mean Z-score profile for the last 150 positions of *H. sapiens* proteins under GO slim cellular component ontologies in reference to column random model**. This heat map represents position-wise Z-score of predicted disordered residues for the last 150 positions near the C-terminal region of *H. sapiens* proteins under each GO slim cellular component ontology. In each functional category, Z-scores for predicted disordered residues were calculated considering proteins (more than 200 residues in length) under that category in reference to their corresponding column random sequences. Here rows represent the positions along the protein sequence and columns represents Z-score coded in color scheme (color legend). Only the ontologies with more than 100 proteins were shown. GO, gene ontology.

IDPs were shown to have a high level of functional specificity compared to ordered globular proteins [3], [4], [5], [6]. Therefore one pertinent question may be whether the signal for high protein disorder near the terminal regions of eukaryotic proteins is function-specific? To dig deeper into this aspect, we grouped human proteins according to their gene ontology (GO-slim) functional categories. Considering three broad GO categories, biological process (BP), molecular function (MF), and cellular component (CC), we found 123 GO-slim functional terms with at least 100 proteins. Next, we compared position-specific disorder scores of proteins under each of these GO-slim terms with their corresponding terminal residue conserved random sequences and column-wise random sequences, specifically generated for each GO category (see Material and methods). Proteins associated with most of these terms showed a preference for high disorder near the N- and C-terminal regions. For instance, ~78% (51 out of 65) of BP terms, ~71% (25 out of 35) of MF terms, and ~69% (16 out of 23) of CC terms showed a moderate to strong preference for high protein disorder near the N-terminal regions, compared to terminal residue conserved random sequences. The exceptional cases in which we found either negative or relatively weaker signals were associated with different types of metabolic and developmental processes for BP, different types of enzymatic functions such as helicase, isomerase, oxidoreductase, and ligase for MF, and protein extra-cellular matrix, ribosome, Golgi apparatus, *etc*. for CC (Figure 4, Figures S34–S44). Probable explanations for this pattern are discussed in discussion. When we compared against column-wise random sequences, we did not find any significant trend near the terminal regions but noticed positive Z-scores throughout the considered regions in most of the functional terms. These results suggest that the trends reported in the aforementioned section are generally not specific to proteins belonging to any particular functional category; however, the strength of the signal (Z-score) may not be at the same level in all such groups.

### Highly and lowly expressed genes show species-specific trends

**Supplementary figure S45:**
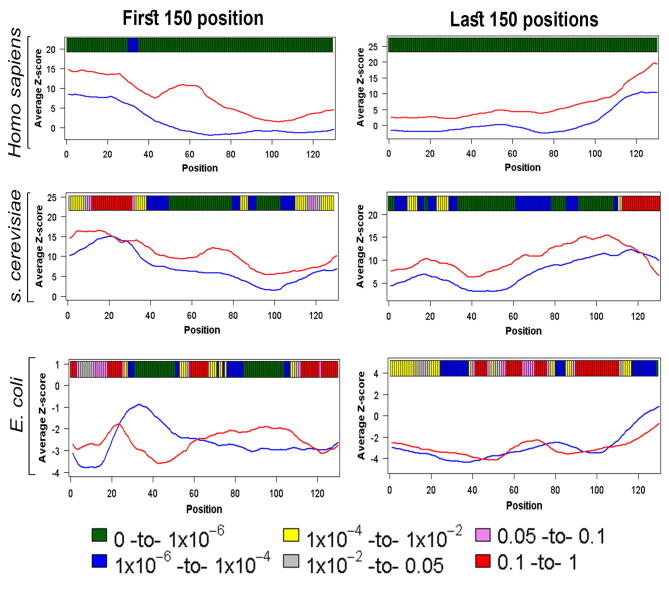
**Comparison of Z-score profile between highly and lowly expressed genes terminal residue conserved random model**. This figure shows the position-wise (first and last 150 positions) Z-scores for predicted disordered residues of proteins encoded by highly and lowly expressed genes of three organisms *H. sapiens*, *E. coli*, and *S. cerevisiae*. For each group of proteins, at each position, we first calculated Z-scores for predicted disordered residues with respect to their terminal residues conserved random sequences then compared Z-scores between highly and lowly expressed groups. Here blue line stands for Z-scores of highly expressed proteins and red line stands for Z-scores of lowly expressed proteins. To access statistical significance of the difference we compared Z-scores between highly and lowly expressed genes considering sliding window of 21 residues starting from the first positions towards the end position with an interval of 1 position. Z-scores were compared window-wise and *P* values were calculated for each such window. Here we plotted the mean Z-score (and associated *P* values) of highly and lowly expressed group in each window positioned at the center of the window. Statistical significance was accessed through Mann-Whitney U test following non-parametric distribution. *P* values were shown in color codes at the top of Z-scores.

**Supplementary figure S46:**
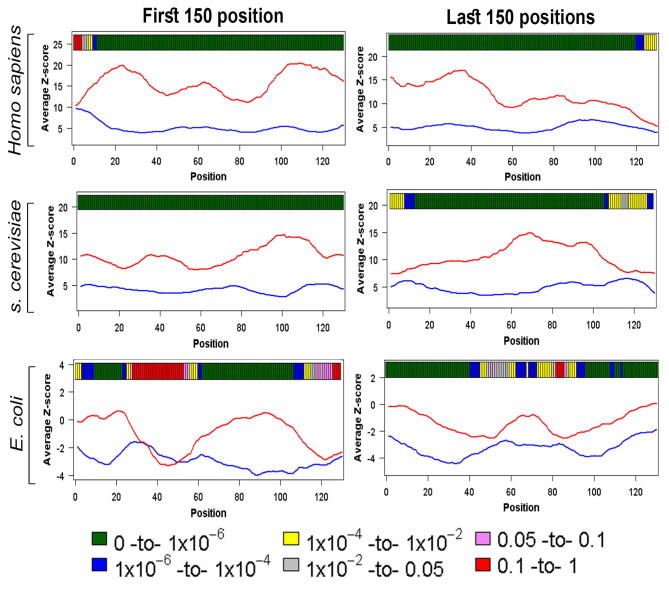
**Comparison of Z-score profile between highly and lowly expressed genes column random model**. This figure shows the position-wise (first and last 150 positions) Z-scores for predicted disordered residues of proteins encoded by highly and lowly expressed genes of three organisms *H. sapiens*, *E. coli*, and *S. cerevisiae*. For each group of proteins, at each position, we first calculated Z-scores for predicted residues with respect to their column random sequences then compared Z-scores between highly and lowly expressed groups. Here blue line stands for Z-scores of highly expressed proteins and red line stands for Z-scores of lowly expressed proteins. To access statistical significance of the difference we compared Z-scores between highly and lowly expressed genes considering sliding window of 21 residues starting from the first positions towards the end position with an interval of 1 position. Z-scores were compared window-wise and *P* values were calculated for each such window. Here we plotted the mean Z-score (and associated *P* values) of highly and lowly expressed group in each window positioned at the center of the window. Statistical significance was accessed through Mann–Whitney *U* test following non-parametric distribution. *P* values were shown in color codes at the top of Z-scores.

IDPs were shown to be expressed at lower levels than well-structured globular proteins [29], [30]. Therefore, we found it interesting to analyze whether the trends we observed near the terminal regions of eukaryotic and prokaryotic proteins vary according to their gene expression levels. We compared the Z-scores (deviation of disorder scores between real and random sequences) between the proteins encoded by highly and lowly expressed genes of three species, *H. sapiens*, *S. cerevisiae*, and *E. coli*, using high-throughput gene expression data. In each of these species, Z-scores were computed separately for highly and lowly expressed proteins using both their terminal residue conserved and column-wise random sequences as reference (see Material and methods). When we compared Z-scores obtained in reference to terminal residue conserved random model, lowly expressed proteins of *H. sapiens* and *S. cerevisiae* showed higher Z-scores than highly expressed proteins near both terminal regions (Figure S45). However, in *E. coli* we did not find a clear difference in Z-scores between these two groups of proteins. When we compared Z-scores obtained in reference to column-wise random sequences, we found similar results (Figure S46).

### Regions showing enrichment of disordered residues in eukaryotic proteins are also enriched with disordered binding sites

**Figure 5:**
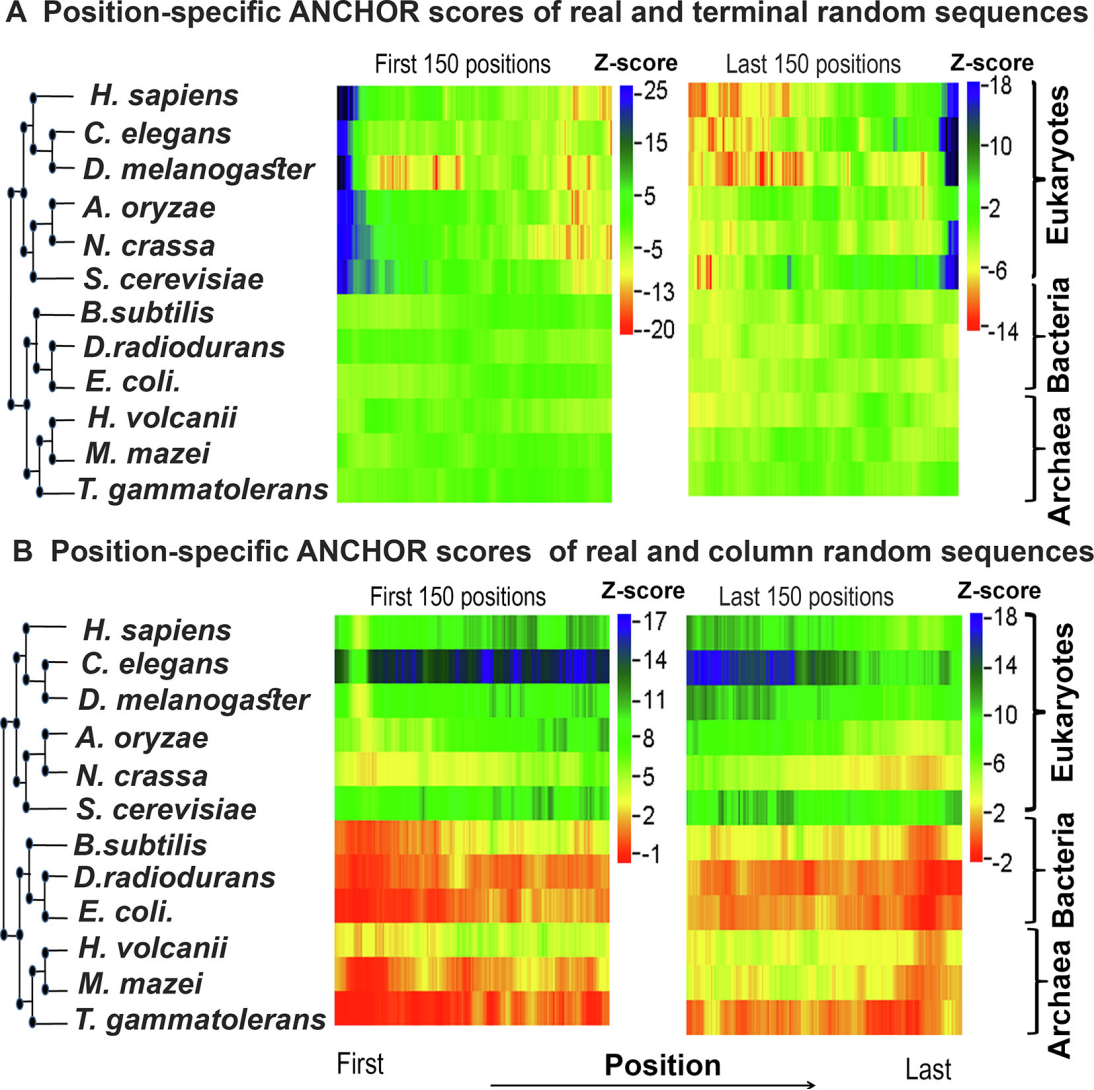
Z-score profile for the position-specific comparison of ANCHOR predicted protein binding residues This figure shows the extent of protein binding sites within disordered regions between real and random protein models for the first 150 and last 150 positions of 12 organisms considered in this study. **A.** Z-scores were calculated by comparing predicted ANCHOR residues of real and terminal residue conserved random sequences of each species. **B.** Z-scores were calculated by comparing predicted ANCHOR residues of real and column-wise random sequences of each species. Z-scores were coded in color scheme (color legend). Here positive Z-score indicates enrichment of ANCHOR predicted protein binding sites in naturally occurring sequences while negative Z-score indicates the reverse. Organisms are arranged according to their mid-point rooted species tree retrieved from NCBI taxonomic database with the help of their species taxonomic identifier.

To test whether disordered residues near the terminal regions of native eukaryotic proteins have any role in protein–protein interactions, we searched for potential interaction sites within those regions using ANCHOR [31]. ANCHOR predicts probable interaction sites within disordered regions and provides an unbiased estimate of the interaction potential conferred by the disordered residues [31]. As shown in Figure 5A and B, eukaryotic proteins generally have more protein–protein interaction sites near the terminal regions compared to the corresponding terminal residue conserved random sequences, while no such trend is observed in prokaryotes. When compared with column-wise random sequences, we found consistently higher Z-scores for predicted ANCHOR residues throughout the examined regions in eukaryotes. Generally, these results are consistent with the position-specific disorder profile of eukaryotic and prokaryotic proteins near the terminal regions. These results may indicate that disordered residues are preferred specifically near the terminal regions of eukaryotic proteins in order to promote protein–protein interactions.

## Discussion

One of the major goals of protein structural biology is to understand the structural and functional characteristics of IDPs. Extensive research over the past few decades, reviewed in references [1], [2], [3], [4], [5], [6], has improved our understanding of these proteins. However, to date, many fundamental issues are not clearly understood. How nature has shaped the disorder potential of naturally occurring eukaryotic and prokaryotic proteins is one of those elusive unresolved questions. Considering naturally occurring proteins from several model organisms and comparing them to artificially generated random protein models, we sought to understand whether there is any preference for disorder residues in the native proteins compared to random expectations.

We generated three kinds of random models. First, to compare proteome-wise disorder content between real and random sequences of each species, we considered a general random model that preserves the overall amino acid composition and length of the proteins but not the order of the amino acids (length conserved random sequences). In addition, we used two other random models to account for the effects of biased amino acid composition near the terminal regions of proteins. One is based on random shuffling only between the terminal residues, and the other maintains overall frequencies of amino acids at any particular position of real proteins and hence is expected to preserve the features induced by the biased distribution of amino acids.

First, we compared proteome-wide protein disorder content between real and random sequences in each of the 12 selected organisms. Based on a small number of proteins (mainly short peptides collected from UniProt Reference Clusters database), previously Yu et al. [23] suggested that high protein disorder in natural sequences is a general evolutionary trend. By contrast, in this systematic analysis, we compared the disorder content of real sequences of each species with their corresponding length conserved random sequences. This allowed us to investigate the trends species-wise. Our results suggest that depending on the characteristics of the species, natural sequences may have more or less disorder content compared to random sequences. A general pattern emerged from these results is that in eukaryotes, at least for the species considered in this study, naturally occurring proteins are more disordered compared to random sequences, but this is not true in prokaryotes. However, we should note that a number of caveats may explain the observed trends (discussed later). Previously, numerous studies have indicated that eukaryotic proteins are generally more disordered than prokaryotic ones [13], [14], [15]. However, to the best of our knowledge, no study has ever analyzed whether there is any disparity in selection for disordered residues between eukaryotic and prokaryotic proteins as we did in this study. Our results suggest the existence of differential selection for disordered residues which may explain the variation in disorder content between eukaryotic and prokaryotic proteins. Disordered residues were supposed to play a crucial role in the rise of complex eukaryotic organisms [4], [5], [13], [16]. Most of the novel protein functions which appeared early in eukaryotic evolution, such as transcription factors, transmembrane receptors, signaling proteins, intracellular communication, cytoskeletal proteins, and chromatin organization, have elevated level of protein disorder. Considering their importance in higher organisms, it was suggested that the proteomic disorder content of a species is linked with its genomic complexity [4], [13]. Thus, the general preference for disordered residues (compared to random expectations) among eukaryotic proteins which we observed in this study may be an evolutionary relic of the role IDPs played in these organisms.

Next, we looked for regions that may have been selected for high or low disorder within the proteins. An earlier study showed that N-terminal regions of DNA binding homeodomain proteins are generally disordered by nature due to their high net charge [32]. Disordered tails at the N-terminal regions were suggested to be advantageous for the DNA binding proteins to serve as an anchor for high specificity and low-affinity binding (fly-casting mechanism) with cognate DNA molecules [32]. However, there is no general understanding of whether disordered residues are uniformly distributed along the protein or there is any site-specific variation. As with proteome-wise disorder content, we noticed a prominent difference in the observed trends between eukaryotic and prokaryotic proteins. Our study suggests that over the course of evolution, eukaryotic proteins have specifically accumulated a higher than expected fraction of disordered residues near the terminal regions (particularly near the N-termini). However, we did not find such a clear trend in prokaryotic organisms, except in *D. radiodurans* and *H. volcanii*, where we found slightly higher disorder in the first and last few positions as compared to random expectations. Based on our results here we propose that high disorder near the terminal regions, specifically near the N-termini, is not limited to DNA binding homeodomain proteins but is a more general trend in eukaryotic proteins.

Below we discuss possible explanations for these trends.

First, the trends we observed in this study may be a side effect of constraints imposed by unrelated factors. Specifically, since the codons of most of the disorder-promoting amino acids are GC rich, a strong association has been found between genomic GC composition and protein disorder content [16], [17], [26]. Earlier, Ángyán et al. [24] suggested that the disorder potential of *de novo* proteins is a function of the GC content of their coding sequences. To disentangle the impact of GC on the observed disparity in proteome-wide disorder content between the real and random sequences, we compared the disorder content of real sequences pulled from different genomic GC backgrounds with that of their corresponding length conserved random sequences (*i.e*., in GC bins). In most species studied here, we found generally similar trends for the bins as were found for all proteins, suggesting that genomic GC has little impact on the observed trends. Next, we tested whether the trends that we observed near the terminal regions of eukaryotic proteins are caused by selection for high GC at the nucleotide level. Constructing similar random models as we did for proteins, we did not find a common trend between predicted disorder and GC content in most species, suggesting that our results are independent of selection for GC at the nucleotide level. In fact, in many organisms, the GC content at the 5′ end of the mRNA is relatively low (probably due to weak mRNA folding in these regions [33], [34]). Thus, this interaction cannot explain the intra-protein disorder pattern we found here.

Second, the amino acid bias found near the ends of proteins may explain the variation in disorder. Amino acids are not uniformly distributed along the sequences. Specifically, terminal regions were shown to prefer charged residues due to their solvent-exposed and flexible nature [28]. Polar and charged residues are also known to increase the propensity of a protein to be disordered [1], [2], [3]. Thus, the trend that we observed near the terminal regions of eukaryotic proteins may have arisen because of the higher fraction of charged residues selected mainly for the solvent-exposed nature of these regions. However, we did not find any parallel trend of preference for solvent accessibility (compared to random expectation) as we found for disordered residues, which suggests that the observed trend is independent of the solvent-exposed nature of these regions. Moreover, the random models we used to compare the position-specific disorder score were generated in view to preserve the characteristics of terminal regions. The terminal residue conserved random model shuffles amino acids within terminal regions, and the column-wise random model shuffles the amino acids at each position of the native proteins. Thus, the random sequences generated using these two methods are supposed to maintain the regional characteristics of the terminal regions. The high protein disorder we found with respect to these random models implies that our results cannot be explained simply by the charged and surface-exposed nature of the terminal regions.

Third, this enrichment may be partially explained by the lack of selection near terminal regions. A previous study comparing evolutionary rate with protein structure has consistently found that exposed sites are more tolerant of amino acid substitutions and evolve at a higher rate than buried sites [35]. This, in turn, suggests that terminal regions, being solvent-exposed, evolve under weaker evolutionary constraints than the central regions. Thus terminal regions were considered as “evolutionary playgrounds” for the innovation of new functions [36]. This reduced efficacy of selection at the protein termini may have provided the permissive environment for the fixation of disorder-promoting amino acids which are generally associated with high rates of insertions, deletions, and substitutions [7], [8], [10]. This effect may be more specific to eukaryotes because of their lower effective population size compared to prokaryotes [37]. Further, disordered residues in these regions may be less deleterious in eukaryotes than in prokaryotes to facilitate their fixation. Nevertheless, the fact that the amino acid distribution near the ends cannot explain these patterns (as we showed based on our null models) supports the conjecture that lack of selection for certain amino acids is not the only explanation. If there was no selection, we would expect to see a similar pattern in the null models. Moreover, the direction of the results is not always what could be expected based on population genomics considerations. According to the population genomics model [37], disordered residues, if deleterious, are expected to be purged from the genomes of higher effective population size due to their higher efficacy of selection. However, as an example, we found comparable trends near the terminal regions of both *S. cerevisiae* and *H. sapiens* despite wide variation in their effective population sizes. This suggests that reduced efficacy of selection as expected from the perspective of effective population size is not the major cause for the trends we found in eukaryotic genomes.

**Figure 6:**
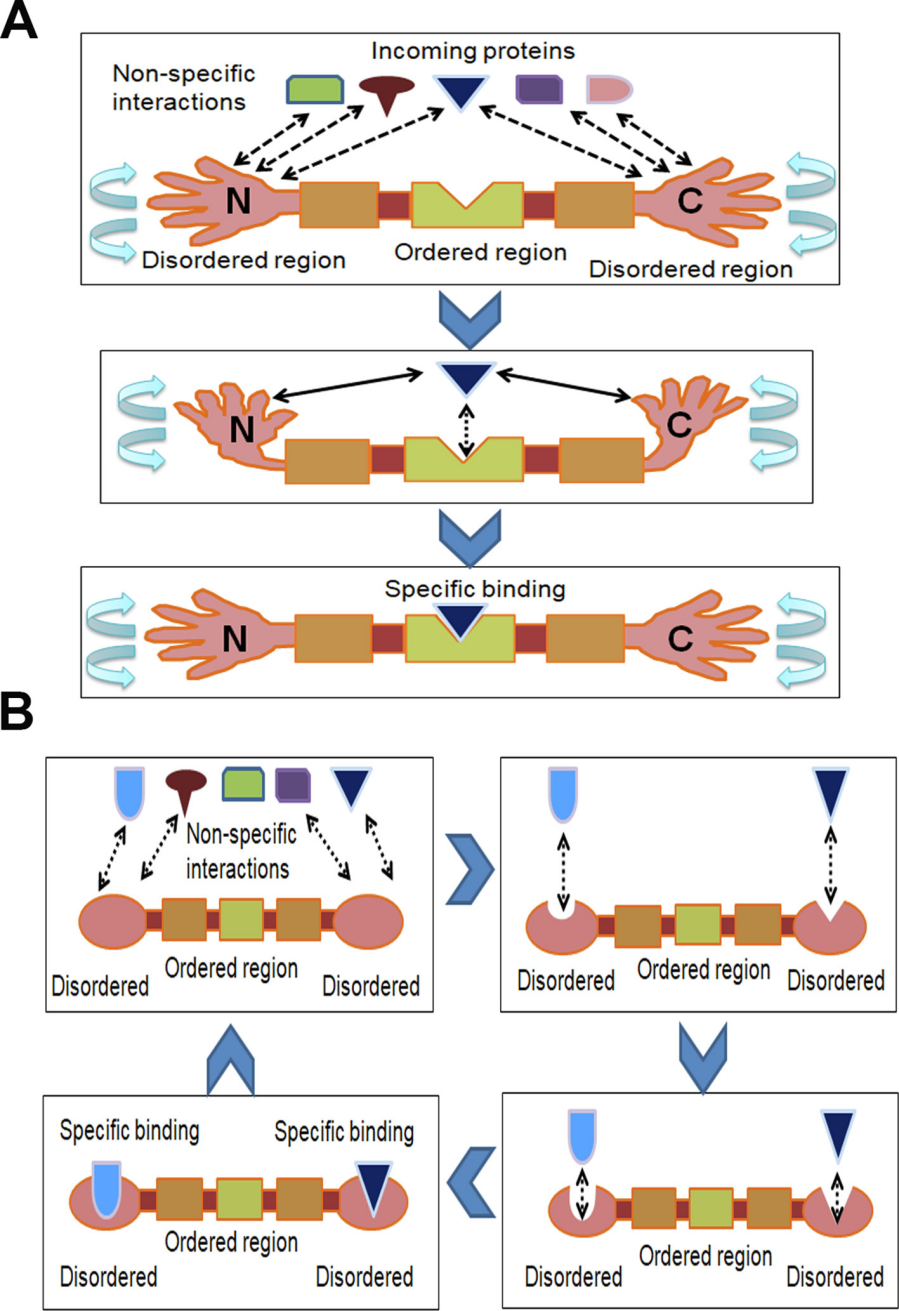
**Potential roles of disordered terminus in protein**–**protein interaction** **A.** Disordered tails of eukaryotic proteins may act like two arms to improve the search for the specific interacting protein partners. Disordered regions bind with the partners with weak and non-specific interactions which may help to mediate more specific interactions. **B.** Being flexible in nature, disordered tails may form binding sites for large number of protein partners.

Fourth, disordered residues may have been selected specifically near the terminal regions of eukaryotic proteins due to functional reasons, especially the higher proximity of protein–protein interactions (as explained in Figure 6A and B). Due to their inherent structural flexibility, disordered residues can form binding sites for a large number of partner proteins [1], [2], [3], [4], [5], [6]. The specific enrichment of disordered residues near protein termini may act as two “arms” which mediate non-specific weak protein–protein interactions that improve the search for the specific interacting protein partner (Figure 6A). To check whether high disorder at terminal regions of eukaryotic proteins has any role in protein–protein interactions, we tested the distribution of disordered binding motifs in real and random sequences in each species. Disordered binding motifs are short stretches of disordered residues that undergo order-to-disorder transition upon binding and are considered crucial in molecular recognition for their binding capacity [5], [11], [12]. Moreover, disordered binding sites may act as a flexible linker for protein–protein interactions [6], [31]. Thus, the higher proportion of disordered binding residues near the terminal regions of eukaryotic proteins may help these proteins to attain structural flexibility for binding promiscuity. In support of this view, disordered N-terminal tails of homeodomain proteins were shown to facilitate DNA search and accelerate specific binding with partner DNA molecules which may be associated with our results [32]. This effect might be more important in the larger, more complex eukaryotic cells rather than in prokaryotic cells.

Fifth, terminal regions of eukaryotic proteins may have evolved to have a higher fraction of disordered residues because of other functional advantages. It is possible that on average the proper functionality of residues or domains near the end of the proteins requires a higher level of disordered residues. Further, the conformational plasticity of disordered residues may be particularly advantageous in the terminal regions compared to core regions. This is evident in the fact that depending on length, 30%–97% of human proteins are predicted to have disordered stretches near their N- and/or C-termini [38]. The diverse roles disordered residues play in protein terminal regions were reviewed elsewhere [39]. In brief, they are advantageous for G-protein-coupled receptors, voltage-gated potassium channels, and ligand binding in the transmembrane region among several others [39]. Disordered regions are also often targeted by several types of PTMs and alternative splicing (AS) [1], [4], [5], [6]. AS and PTMs are important means of generating the structural and functional diversity of eukaryotic proteins without significantly affecting genome size. A higher fraction of disordered residues near the protein termini may have evolved for the functional exaptation in form of sites for AS and/or PTMs, which have been shown to be more prominent in terminal regions and especially in eukaryotic proteins [40].

Sixth, the disparity in protein disorder between eukaryotes and prokaryotes may be related to the differences in their protein folding pathways including the differences in ways how nascent peptide chains acquire structure and the ribosomes of the two domains. Particularly, a possible explanation for the lack of a significant trend of high disorder in prokaryotic organisms may lie in their growth kinetics. Maximization of growth rate is a fundamental aspect of prokaryotic biology [41]. Most bacteria grow extremely fast (generation time usually ranges from a few minutes to several hours) [41], while a typical eukaryotic (human) cell takes about 24 h to divide [42]. To achieve their higher growth rates, prokaryotes are under strong selection to adopt several strategies such as increasing the speed and efficiency of their replication and translation [41]. Recent experiments suggest that the speed with which nascent peptides emerge from the ribosome is an important parameter that determines subsequent folding and can also alter the final conformation of the protein. When peptides are synthesized, nascent peptides pass through the negatively charged ribosomal exit tunnel. Therefore, positively charged residues were thought to retard the protein translational rate [43]. Later, considering more diverse datasets, it has been shown that it is not only positively charged residues, but charged residues (positive or negative) in general, may cause stalling [44]. This may have a positive effect on fitness at the 5′ end of the mRNA [45], [46]. Disordered regions, in general, are enriched with polar and charged residues [1], [2], [3]. Hence, a high amount of disordered residues, especially near the rate-limiting initiation site (in N-terminal regions), may be detrimental to the higher growth rates of prokaryotic organisms; specifically, it is possible that the relationship is direct that disordered residues may increase interaction with the ribosomal exit tunnel. Therefore, it is conceivable that prokaryotes will tend to use fewer disordered residues compared to random expectation, and especially near the protein termini.

We discussed some probable explanations for the enrichment of disordered residues near the protein ends in eukaryotes; however, further studies and experiments will be needed to fully understand their relevance.

A position-dependent relationship has been proposed between protein disorder and function, suggesting that the relative position of the unstructured region within a protein provides clues regarding its functionality [47]. Specifically, proteins forming different kinds of ion channels and those involved in transcription factor activation or repression were supposed to have a higher fraction of disordered residues near their N- and C-termini, respectively, compared to proteins related to transcription regulation, RNA pol II transcription, and DNA binding processes, *etc.*, which contain disordered regions near the interior [47]. However, it is not clear from that study if this trend is caused by selection for high or low disorder in these functional classes. We investigated whether high disorder near the N- and C-termini of eukaryotic proteins is specific to any functional classes. Considering 123 general functional annotations from the GO-slim database for human proteins, we showed that except for a few specific functional classes (mainly related to different types of enzymatic functions and metabolic processes), high disorder at the terminal regions is a common feature of human proteins belonging to most of the other functional classes. Generally, these results are in line with previous observations which suggest that disordered residues are specifically enriched among functional classes such as signaling, transcription, cell division, apoptosis, PTM, and various regulatory processes while being depleted among proteins involved in enzymatic and catalytic functions [3], [4], [5], [6]. Disordered regions were ascribed to be advantageous for the above-mentioned enriched processes because of their functional prerequisites such as high-specificity and low-affinity binding, ease of regulatory control, which cannot easily be achieved with structured proteins [3], [4], [5], [6]. However, those studies mainly considered the disorder content of the entire protein without looking for any site-specific signature. Here we show each of those functional classes bears a specific signature for high or low disorder near the protein termini.

High expression of IDPs was assumed to be detrimental for cell survival because of their harmful effects when over-expressed [11], [29], [30]. Since a high concentration of IDPs may lead to several disease conditions, cells were proposed to develop several mechanisms to keep their expression levels below a certain threshold [11], [29], [30]. This notion is supported by the observation that in higher organisms such as *H. sapiens* and *S. cerevisiae*, IDPs are generally expressed at lower levels than the globular proteins [29], [30]. This may suggest that in eukaryotes highly expressed genes would show weaker selection for disordered residues than lowly expressed genes. Indeed, when we compared Z-score for predicted disordered residues between proteins encoded by highly and lowly expressed genes, the lowly expressed group showed a stronger signal (higher Z-score) in *H. sapiens* and *S. cerevisiae* but not in *E. coli*. Previously, a weak positive correlation was noted between gene expression level and protein disorder in *E. coli*, suggesting that since prokaryotes encode relatively fewer disordered residues expression level may not be a strong burden for IDPs in these organisms [48]. In accordance, in *E. coli* we did not find a prominent difference in Z-score between the highly and lowly expressed groups, which may suggest that the trends that we observed at the protein level are independent of gene expression at the transcript level in prokaryotes.

In conclusion, in this study, we compared disorder scores between real and random sequences of several eukaryotic and prokaryotic organisms. Our study suggests that disordered residues are preferred in eukaryotic proteins over random expectations and this preference is stronger near the protein termini. Prokaryotic proteins, however, show either no or weak preference for disordered residues. Based on these observations we discussed several explanations. However, we would like to re-emphasize that most of these are predictive in nature. Therefore, further experiments are needed to understand the causes and consequences of the trends shown here. Moreover, in this analysis, we mainly compared protein disorder between the two major groups, eukaryotes and prokaryotes, where the observed differences are distinct and very clear. However, there is wide variation in the strengths of selection among the genomes within each domain (eukaryotes or prokaryotes). Each organism may specifically tailor the level of disordered residues in its proteome according to its functional and environmental prerequisites [13], [14], [15]. Therefore, one important direction for future research could be to explore the factors responsible for this intra-domain variation in protein disorder.

## Materials and methods

Here we briefly described the experiments done in this study. Detailed material and methods can be found in File S1. In this study, we considered naturally occurring protein sequences of 12 model species, including six prokaryotes (three bacteria: *B. subtilis*, *E. coli*, and *D. radiodurans*; three archaea: *M. mazei*, *H. volcanii*, and *T. gammatolerans*) and six eukaryotes (*H. sapiens*; one insect: *D. melanogaster*; one worm: *C. elegans*; three fungi: *S. cerevisiae*, *A. oryzae*, and *N. crassa*). In each species, proteins containing ambiguous amino acids (B, J, O, U, X, and Z) and internal stop codons or partial codons in their corresponding coding sequences were removed. Next, for each species, we generated three sets of random protein sequences from the real protein sequences of that species. Potential disordered residues within the real and randomly generated protein sequences were estimated following consensus approach based on the prediction from a set of well-known disorder prediction algorithms. Then we compared the overall disorder content as well as position-wise disorder score (based on consensus approach) of real protein sequences of each species with their corresponding random protein sequences thorough the Z-score approach. To check the impact of confounding factors on the protein disorder scores of real and random sequences, we considered several factors such as genic GC content, protein length, proximity to splice junction, and effect of solvent accessible surface areas. Here we also compared potential protein–protein binding sites within disordered regions between real and random sequences of each species following Z-score approach.

## Data availability

Results of disordered prediction as well as GC content and predicted solvent accessibility for all real and random datasets can be obtained from the authors upon request.

## CRediT author statement

**Arup Panda:** Conceptualization, Methodology, Validation, Formal analysis, Data curation, Writing - original draft, Visualization. **Tamir Tuller:** Supervision, Methodology, Formal analysis, Data curation, Conceptualization, Project administration, Writing - review & editing, Funding acquisition. All authors read and approved the final manuscript.

## Competing interests

The authors declare that they have no competing interests.

## Supplementary material

Supplementary File S1 Supplementary materials and methods.

Supplementary Table S1 List of genomes used in the analysis.

Supplementary Table S2 Table of correlation between the results of different disorder prediction algorithms and experimental annotation.
