# Supplementary material for: Exploring Potential Signals of Selection for Disordered Residues in Prokaryotic and Eukaryotic Proteins
Source: Genomics Proteomics Bioinformatics. 2020 Dec 18;18(5):549–64. doi: 10.1016/j.gpb.2020.06.005 (PMC8377245; doi:10.1016/j.gpb.2020.06.005)
Supplement: Supplementary File S1 — Supplementary materials and methods. [file mmc1.docx]

**File S1 Supplementary materials and methods**

**Data collection**

For our study we considered 12 model species, six prokaryotes and six eukaryotes: *H. sapiens*, one insect: *D. melanogaster*, one worm: *C. elegans*, three fungi: *S. cerevisiae*, *A. oryzae,* and *N. crassa*, three bacteria: *B. subtilis*, *E. coli*, *D. radiodurans,* and three archaea: *M. mazei*, *H. volcanii*, *T. gammatolerans*. All these organisms are listed in Table S1 with details of their characteristics such as their accession number, strain description, genome size, and number of predicted proteins, *etc*. We choose these organisms following Faure et al. [1] considering our objective to get a general overview in the eukaryotic and the prokaryotic domain. Except for *H. sapiens* (downloaded from Ensembl v-90 [2]) and *S. cerevisiae* (downloaded from the *Saccharomyces* Genome Database [3]) protein coding sequences and complete proteomes of all the remaining species were retrieved from the national center for biotechnology information (NCBI) Reference Sequence database (RefSeq) [4] (release 88) (last accessed 6^th^ March’ 2018). Here we choose our genomes mostly from the NCBI RefSeq database because the protein sequences in RefSeq are non-redundant, well-annotated and explicitly linked with their nucleotide sequences [4]. To ensure the quality of genomes, RefSeq imposes strict evaluation criteria and corrects annotation manually when necessary [4]. Further, RefSeq considers spliced variants only when there is experimental evidence against their full-length nature thus only a minimal set of splice variants are included in this database. Therefore, chances that our results may be biased by the over-representation of some specific splice variants are very low. On the other hand, Ensembl is also a database of high quality genomes [2], however it includes a huge number of transcript variants. Therefore, to remove redundancy in our dataset (human) we took the longest isoform when we found more than one isoform for a gene.

For a species, if genomes of multiple strains are available we choose the strains annotated as the reference or representative strain. In each species, proteins containing ambiguous amino acids (B, J, O, U, X, and Z) and internal stop codons or partial codons in their corresponding CDS sequences were removed and only proteins more than 50 amino acids in length were considered for proteome-wide disorder prediction.

**Generation of random models**

To serve specific purposes of our study we generated three kinds of random protein models for each species. First, to compare the protein-wise disorder score between real and random sequences we generated random sequences preserving the overall amino acid composition and length of each real protein (designated as length conserved random model). For this purpose, we randomly shuffled the amino acids of each real protein 10 times (total number of length conserved random sequences of each species = 10 × number of real sequences). Next, to compare position-wise disorder score between real and random sequences we looked for random models that would preserve position-specific amino acid characteristics. For this, we at first generated random sequences (designated as terminal residues conserved random model) by shuffling the amino acids at the N and C-terminals of each real protein. This random model is analogous to our length conserved random model however here we considered only the first and last 200 amino acids (of real proteins more than 200 amino acids in length) for random shuffling. As with our length conserved random, we generated 10 such random sequences for each terminal corresponding to each real protein (total number of terminal residue random sequences for each species = 10 × 2 × number of real sequences). Next, we aligned the naturally occurring proteins of each species from both ends (*i.e.,* from the first amino acid position to the end of the protein and from the last amino acid position to the beginning of the protein) and shuffled the amino acids in each position of the alignment (up to 200 positions) column-wise. Thus it preserves the overall amino acid frequencies at each position of real proteins. To account for the variation in protein length here we considered up to 200 positions from both ends considering only proteins more than 200 amino acids in length. For each species, we generated 200,000 (100,000 for each terminal) such random sequences (designated as column-wise random model). All these three types of random models are illustrated in Figure 1. Here it is noteworthy that for the generation of random sequences (model 2, 3) we considered up to 200 positions from both ends, however, position-specific disorder scores were calculated up to 150 positions (discussed in the next section).

**Prediction of disordered residues**

*Consensus approach 1*

Ordered or disordered status of each residue in each protein was estimated by consensus-based approach. At first, the per-residue disorder scores were predicted using four different disorder prediction algorithms namely IUPred [5,6], VLS2B [7], MoreRONN [8], and DisEMBL [9]. If a residue is predicted as disordered (or predicted disorder score > 0.5) by three of these four algorithms then the residue is considered as a disordered residue. We choose these algorithms because these algorithms do not use any homology profile thus were expected to give an unbiased estimate of disorder scores when applied to random protein models [10]. Among these, IUPred predicts disorder scores based on inter-residue interactions probabilities (estimated through pair-wise interaction energies) [6]. There are two different variants of IUPred, IUPred-S: optimized for short disordered regions and IUPred-L: optimized for long disordered regions [6]. Here we used IUPred-L, which was shown to perform better than IUPred-S in general benchmark datasets [11]. VLS2B, a neural network based algorithm gives a weighted average of disordered scores from two different disorder prediction algorithms trained on short and long proteins [7]. VSL2B was shown to be among the top performing algorithms when compared with 16 other disorder predictors with a benchmark dataset of 514 experimentally verified disordered proteins [12]. MoreRONN is the upgraded version of well known neural network based disordered predictor RONN [8], which was considered as the best of all other 9 disorder prediction algorithms in CASP6 assessment [13]. The standalone version of MoreRONN (https://app.strubi.ox.ac.uk/MoreRONN/) was kindly provided by the RONN [8] developing team prior to their publication. DisEMBL is an artificial neural networks based algorithm which is trained on three different types of disorder dataset: coils, hot loops and missing coordinates in X-Ray structure [9]. Here we considered the version of DisEMBL which defines disordered based on missing coordinates in X-Ray structure (DisEMBL-465). Considering segment overlap measure (SOV), DisEMBL-465 was suggested as the best of 10 other disorder prediction algorithms in a recent assessment [14]. All these algorithms were run locally using default parameter settings. Most of these disorder prediction algorithms use sequence context (*i.e.,* neighborhood) therefore for prediction of disordered residues of random protein models generated from truncated sequences (model 2, 3) we utilized extra 50 residues from both ends *i.e.,* for disorder prediction we used sequences of 200 residues however considered up to first 150 positions in all subsequent disorder calculation.

*Consensus approach 2*

Except for MoreRONN [8] (upgraded in 2017), all other algorithms like IUPred [5,6], DisEMBL [9] and VSL2B [7] were published more than a decade ago. Therefore, during the revision process, we were suggested to check our results with comparatively newer disorder prediction algorithms. To further validate our results here we considered three algorithms which are single sequence based, not used in our initial analysis and published or upgraded in a recent time frame. In the way of our search for new algorithms, we noticed that a newer version of IUPred (IUPred 2A) has been published last year [15]. Considering the fact that we have a very heterogeneous dataset (real and random proteins) which may have long or short disordered regions we decided to use both the verities of IUPred2A *i.e*., short and long. We found another well known single sequence based disorder predication algorithm Espritz, which is published few years back [16]. To predict disordered regions, ESpritz employs bi-directional recursive neural network-based machine learning method and was tested to perform better than most of the other disorder prediction algorithms [16]. Based on the training dataset, Espritz has three variants (i) Espritz-N (trained on NMR mobility dataset), (ii) Espritz-X (trained on proteins with known X-ray crystal structure), and (iii) Espritz-D (trained on proteins from DisProt database) [16]. Here we considered all these three variants with the option to maximize accuracy threshold (best Sw score) and ignoring PSI blast option. Recently a new single-sequence based disorder prediction algorithm was developed which was suggested to be more accurate than sequence profile based prediction algorithms for proteins with limited number of homologous [17]. This algorithm knows as SPOT-Disorder-Single is based on an ensemble of current state-of-art-neural networks [17]. Here we downloaded its standalone version and run with default settings. To determine consensus between these algorithms first we considered each variant as independent predictors and calculated their majority vote *i.e.,* a residue was considered to be disordered if at least 4 of 6 algorithms (2 variants of IUPred2A, 3 variants of Espritz and SPOT-Disorder-Single) suggested so. Next, we calculated consensus between three algorithms IUPred2A, Espritz and SPOT-Disorder-Single considering their equal weightage. For this, we first inferred ordered or disordered status of each residue by integrating prediction from the different variants of IUPred2A and Espritz into a final prediction (here denoted as IUPred2A-meta and Espritz-meta). For IUPred2A-meta, we considered a residue as disordered if average disorder score predicted by its two variants (long and short) > 0.5. For Esprtiz-meta, we considered mutual agreement between 2 of its 3 variants. The prediction from IUPred2A-meta and Espritz-meta is then combined with that of SPOT-Disorder-Single and the final consensus was calculated considering their majority vote. Overall, we found very similar results by both these ways of calculating consensus; therefore, here we presented results obtained by one of these methods (consensus calculated considering equal weightage of each algorithm).

We tested the performance of the algorithms used for this study on experimentally known disorder protein dataset (check Table S2 for details). Here we measured correlations between the proportions of disordered residues predicted by each of these algorithms in terminal regions (up to the first and last 150 residues) of these proteins with that calculated based on experimental annotation. Strong correlations between the predicted and experimental values for most of disorder prediction algorithms suggest that these algorithms can reliably predict disorder residues in test proteins (Table S2).

**Calculation of protein disorder content and position-wise disorder scores**

To get relative estimates of structural disorder in the native sequences of each species here we first compared their disorder content with that of length conserved random sequences. Overall disorder content of proteins in each group (real vs. random of each species) was calculated as the average percentage of disordered residues (predicted by a consensus-based approach) in the proteins of that group. To reduce the effects of short flexible loops in disorder prediction [18], we considered another measure of protein disorder content, the percentage of dis_30 residues, calculated as the percentage disordered residues only in long disordered segments (more than 30 or more consecutive disordered residues).

Next, in order to check whether there is any site-specific selection for high or low disorder in naturally occurring protein sequences, we compared the position-specific disorder scores (up to first and last 150 residues) of real sequences with that of two kinds of random protein models (random models 2, 3) which were specifically generated to take into account for the position-specific bias in amino acid composition. To calculate position-specific disorder scores of real proteins, naturally occurring sequences of each species were lined up according to their start position and again according to their end position. Position-specific disorder score of real proteins at any position ‘i’ was calculated as the fraction residues predicted as disordered residues (by consensus-based approach) at that position (‘i’) to the total number of sequences in the alignment (here ‘i’ equal to 1 to 150 from both ends). Position-specific disorder scores of random sequences were calculated in the same way as the real sequences. However, for the estimation of Z-score, we divided the terminal residue conserved and column random sequences of each species into 100 randomized proteomes and estimated position-specific disorder scores for each such randomized proteome. For each position “i” of each such random model we then computed the mean and standard deviation of position-wise disorder score over the 100 randomized proteomes.

We then compared the position-wise disorder score of real protein sequences with that of random protein models at the corresponding position thorough Z-score defined as

*Z-score= (Disorder score of real sequences at any position - mean disorder score of randomized* *proteomes at that position)/standard deviation of the randomized proteomes at that position*

At any particular position, a positive Z-score indicates the enrichment of disordered residues in the naturally occurring sequences while a negative Z-score indicates its enrichment in random sequences. Statistical significance of the difference is accessed through *P*-values calculated via integrating over the relevant area of the related normal distribution. Here *P-*values were calculated following two-tailed distribution considering the probability that disordered scores of real sequences can be higher than that of random sequences or vice-versa.

**Calculation of GC content and generation of random models**

GC content of protein-coding sequences was calculated using CodonW (http://codonw.sourceforge.net). To compare disorder content of real and random proteins in GC bins, proteins were grouped according to the GC content of their coding sequences. To encompass the maximum number of sequences while maintaining a uniform GC variation GC bins were chosen in steps of 10% GC variation whereas starting points are adjusted according to the distribution of genic GC content of each species. For instance, in eukaryotes, GC bins were chosen starting from 40% genic GC except for two low GC genomes *D. melanogaster* and *N. crassa* where GC bins were set starting from 30% genic GC. In prokaryotes, GC bins were chosen starting from 30% genic GC except for two high genomes *H. volcanii* and *D. radiodurans* where the minimum threshold for GC bins was set at 50% genic GC. GC bins defined by this way include > 99% of protein-coding sequences in each species. For position-wise comparison of GC content between real and random sequences we generated random nucleotide models analogous to terminal residue conserved and column random protein models (models 2, 3). For terminal residue conserved nucleotide model, we shuffled the codons in the first and last 450 positions of coding sequences corresponding to first and last 150 positions of protein sequences. For column random model we aligned the codons of real sequences from both ends and then shuffled the codons in each position of the alignment. Z-score and *P*-values were calculated by similar approach as utilized for comparing disorder score.

**Categorization according to gene ontology (GO) functional annotations and gene expression level**

GO slim functional annotations (total 130 categories) of human protein-coding genes were retrieved from Ensembl 90 [2]. GO slim categories with less than 100 genes were removed finally 124 categories were considered. Next, we compared the position-specific disorder score of real sequences under each such category following Z-score approach as described previously with two kinds of random protein models (model 2 and 3). For terminal residue conserved model (model 2) we considered the random sequences corresponding to real sequences under each GO slim category and column random sequences (model 3) were generated separately for each GO slim category from the alignment of proteins in that category. For the expression level of human proteins at the transcript level, we considered high-throughput RNA-seq gene expression data of Uhlén et al. [19]. In this dataset FPKM (fragments per kilobase of exon model per million mapped reads) level of 20,344 genes across 32 normal human tissues has been provided. From this dataset, we retrieved tissue-averaged gene expression level of 18,497 of 19,961 human proteins considered in this study. Gene expression level of *E. coli* (4022 of 4064 proteins) and *S. cerevisiae*, (5122 of 5848) proteins were retrieved from Geo Gene Expression Omnibus [20] (https://www.ncbi.nlm.nih.gov/geo/query/acc.cgi?acc=GSE67218) and publication of Ghaemmaghami et al*.* [21] respectively. For our comparative study, in each of these three species, we compared the Z-scores of predicted disorder between the top and bottom 20% of proteins sorted according to their gene expression level. Z-scores of each group of high and low expressed proteins were calculated in reference to their corresponding terminal residue conserved random sequences and column random sequences. Column random sequences were generated for each group of highly and lowly expressed proteins separately from the alignment of proteins in that group.

**Prediction disordered binding sites with ANCHOR**

To identify the residues along the proteins those may be important for protein binding we considered ANCHOR [22]. ANCHOR predicts the elements within disordered regions those play crucial roles in molecular recognition and protein-protein interactions. To predict such residues, it utilizes the same general principle (pair-wise inter-residue interaction energies) as the IUPred disorder prediction algorithm [22]. However, the basic difference between these two algorithms is that while IUPred predicts all the potential disordered regions within a protein, ANCHOR predicts the disordered regions which could act as potential protein-protein binding sites [22]. Thus the scores predicted by ANCHOR were suggested to be independent of IUPred algorithm [22]. Although these regions are usually very short in length and are associated with very high false positive detection rate, ANCHOR was shown to achieve an accuracy rate of average ~ 67% when tested with different benchmark datasets [22]. For each position (first and last 150 positions) we compared the proportion of disordered binding residues predicted by ANCHOR between real and random protein sequences (random model 2, 3) of each species following Z-score approach as described for position-specific disorder score. The proportion of disordered binding regions in each position was calculated as the number of residues predicted as disordered binding regions at that position divided by the total number of sequences in the dataset.

**Prediction of solvent accessible surface area**

Solvent accessible surface area (ASA) of each protein in our datasets was predicted using the standalone version of SPIDER3-Single algorithm [23]. SPIDER3-Single predicts the extent of solvent exposure of each residue within a protein along with several other structural information based on sequence information only *i.e.,* without using homology profile. This algorithm uses similar types of neural networks as SPOT-Disorder-Single [23], however, considers different sets of input features. As tested by the authors, this algorithm is more accurate than other state-of-art ASA prediction algorithms such as ASAquick or profile-based SPIDER3 [23]. Here we calculated Z-score for predicted ASA following the steps as described for disorder prediction. For the sake of brevity, this test was done only for three species *H. sapiens*, *E. coli* and *S. Cerevisiae* using the same real and random (terminal residue conserved and column random) datasets as used for disorder prediction.

**Statistical analyses**

All statistical tests were performed using R [version] (The R Project for Statistical Computing [24]). We compared the percentages of disordered residues (not normally distributed) between real and random datasets using two samples “Wilcox.test” (commonly known as the Mann-Whitney U test) function of R. Following non-parametric distribution, all the correlation analyses were performed using Spearman’s Rank correlation test and significant levels were denoted with *P* values.

**References**

[1] Faure G, Ogurtsov AY, Shabalina SA, Koonin EV. Role of mRNA structure in the control of protein folding. Nucleic Acids Res 2016;44:10898−911.

[2] Zerbino DR, Achuthan P, Akanni W, Amode MR, Barrell D, Bhai J, et al. Ensembl 2018. Nucleic Acids Res 2018;46:D754−61.

[3] Cherry JM. The *saccharomyces* genome database: a tool for discovery. Cold Spring Harb Protoc 2015;2015:pdb.top083840.

[4] Pruitt KD, Tatusova T, Maglott DR. NCBI reference sequences (RefSeq): a curated non-redundant sequence database of genomes, transcripts and proteins. Nucleic Acids Res 2007;35:D61−5.

[5] Dosztanyi Z, Csizmok V, Tompa P, Simon I. The pairwise energy content estimated from amino acid composition discriminates between folded and intrinsically unstructured proteins. J Mol Biol 2005;347:827−39.

[6] Dosztanyi Z, Csizmok V, Tompa P, Simon I. IUPred: web server for the prediction of intrinsically unstructured regions of proteins based on estimated energy content. Bioinformatics 2005;21:3433−4.

[7] Peng K, Radivojac P, Vucetic S, Dunker AK, Obradovic Z. Length-dependent prediction of protein intrinsic disorder. BMC Bioinformatics 2006;7:208.

[8] Yang ZR, Thomson R, McNeil P, Esnouf RM. RONN: the bio-basis function neural network technique applied to the detection of natively disordered regions in proteins. Bioinformatics 2005;21:3369−76.

[9] Linding R, Jensen LJ, Diella F, Bork P, Gibson TJ, Russell RB. Protein disorder prediction: implications for structural proteomics. Structure 2003;11:1453−59.

[10] Ángyán AF, Perczel A, Gáspári Z. Estimating intrinsic structural preferences of de novo emerging random-sequence proteins: is aggregation the main bottleneck? Febs Lett 2012;586:2468−72.

[11] Sirota FL, Ooi HS, Gattermayer T, Schneider G, Eisenhaber F, Maurer-Stroh S. Parameterization of disorder predictors for large-scale applications requiring high specificity by using an extended benchmark dataset. BMC Genomics 2010;11:S15.

[12] Peng ZL, Kurgan L. Comprehensive comparative assessment of in-silico predictors of disordered regions. Curr Protein Pept Sci 2012;13:6−18.

[13] Jin Y, Dunbrack RL. Assessment of disorder predictions in CASP6. Proteins 2005;61:167−75.

[14] Walsh I, Giollo M, Di Domenico T, Ferrari C, Zimmermann O, Tosatto SC. Comprehensive large-scale assessment of intrinsic protein disorder. Bioinformatics 2015;31:201−8.

[15] Mészáros B, Erdos G, Dosztányi Z. IUPred2A: context-dependent prediction of protein disorder as a function of redox state and protein binding. Nucleic Acids Res 2018;46:W329−37.

[16] Walsh I, Martin AJ, Di Domenico T, Tosatto SC. ESpritz: accurate and fast prediction of protein disorder. Bioinformatics 2012;28:503−9.

[17] Hanson J, Paliwal K, Zhou Y. Accurate single-sequence prediction of protein intrinsic disorder by an ensemble of deep recurrent and convolutional architectures. J Chem Inf Model 2018;58:2369−76.

[18] Light S, Sagit R, Ekman D, Elofsson A. Long indels are disordered: a study of disorder and indels in homologous eukaryotic proteins. Biochim Biophys Acta 2013;1834:890−97.

[19] Uhlén M, Fagerberg L, Hallström BM, Lindskog C, Oksvold P, Mardinoglu A, et al. Tissue-based map of the human proteome. Science 2015;347:1260419.

[20] Barrett T, Wilhite SE, Ledoux P, Evangelista C, Kim IF, Tomashevsky M, et al. NCBI GEO: archive for functional genomics data sets--update. Nucleic Acids Res 2013;41:D991−5.

[21] Ghaemmaghami S, Huh WK, Bower K, Howson RW, Belle A, Dephoure N, et al. Global analysis of protein expression in yeast. Nature 2003;425:737−41.

[22] Dosztanyi Z, Meszaros B, Simon I. ANCHOR: web server for predicting protein binding regions in disordered proteins. Bioinformatics 2009;25:2745−46.

[23] Heffernan R, Paliwal K, Lyons J, Singh J, Yang Y, Zhou Y. Single-sequence-based prediction of protein secondary structures and solvent accessibility by deep whole-sequence learning. J Comput Chem 2018;39:2210−16.

[24] Grunsky EC. R: a data analysis and statistical programming environment - an emerging tool for the geosciences. Computers Geosciences 2002;28:1219−22.
