# Supplementary material for: Exploring Potential Signals of Selection for Disordered Residues in Prokaryotic and Eukaryotic Proteins
Source: Genomics Proteomics Bioinformatics. 2020 Dec 18;18(5):549–64. doi: 10.1016/j.gpb.2020.06.005 (PMC8377245; doi:10.1016/j.gpb.2020.06.005)
Supplement: Supplementary Table S1 — List of genomes used in the analysis. [file mmc2.docx]

**Table S2 Table of correlation between the results of different disorder prediction algorithms and experimental annotation**

| **Algorithm** | **For the first 150 positions** | **For the last 150 positions** |
| --- | --- | --- |
| IUPred | ρ = 0.918; *P <* 1 × 10^-6^ | ρ = 0.768; *P <* 1 × 10^-6^ |
| VSl2B | ρ = 0.952; *P <* 1 × 10^-6^ | ρ = 0.829; *P <* 1 × 10^-6^ |
| DisEMBL | ρ = 0.828; *P <* 1 × 10^-6^ | ρ = 0.51; *P <* 1 × 10^-6^ |
| MoreRONN | ρ = 0.935; *P <* 1 × 10^-6^ | ρ = 0.653; *P <* 1 × 10^-6^ |
| Espritz (D) | ρ = 0.855; *P <* 1 × 10^-6^ | ρ = 0.87; *P <* 1 × 10^-6^ |
| Espritz (N) | ρ = 0.45; *P <* 1 × 10^-6^ | ρ = 0.066; *P =* 4.19 × 10^-2^ |
| Espritz (X) | ρ = 0.923; *P <* 1 × 10^-6^ | ρ = 0.805; *P <* 1 × 10^-4^ |
| IUPred 2A (long) | ρ = 0.916; *P <* 1 × 10^-6^ | ρ = 0.741; *P <* 1 × 10^-4^ |
| IUPred 2A (short) | ρ = 0.799; *P <* 1 × 10^-6^ | ρ = 0.639; *P <* 1 × 10^-4^ |
| Spot disorder Single | ρ = 0.935; *P <* 1 × 10^-6^ | ρ = 0.623; *P <* 1 × 10^-4^ |

*Note*: This table represents the correlation between the proportions of disordered residues predicted by different disorder prediction algorithms with that calculated based on experimental order/disorder annotation. For this test, we calculated proportion of disordered residues up to the first and last 150 positions (separately) of DisProt protein dataset (i) once following order or disorder annotation as suggested in this database based on experimental methods and (ii) then based on the prediction by each of algorithms used for our main analysis. To calculate the proportion of disordered residues position-wise, we aligned the proteins once from start to end and then from end to start. Proportion of disordered residues at any position is calculated as the number of disorder residues (either annotated by DisProt or predicted by algorithm) at that position to the number of proteins in the dataset. Currently, there are 803 sequences in the DisProt database. Here we considered proteins more than 150 residues in length (a total of 604 proteins). Following non-parametric distribution, here we performed Spearman’s Rank correlation analysis and denoted correlation coefficients with ρ and significance level with *P* values.
