# Supplementary material for: Exploring Potential Signals of Selection for Disordered Residues in Prokaryotic and Eukaryotic Proteins
Source: Genomics Proteomics Bioinformatics. 2020 Dec 18;18(5):549–64. doi: 10.1016/j.gpb.2020.06.005 (PMC8377245; doi:10.1016/j.gpb.2020.06.005)
Supplement: Supplementary Table S2 — Table of correlation between the results of different disorder prediction algorithms and experimental annotation. [file mmc3.docx]

**Table S1 List of genomes used in the analysis**

| **Lineage** | **Species name**  **(RefSeq accession number)** | **Genome type** | **Strain/** **Genome assembly** | **Number of real proteins** | **Number of proteins larger than 200 residues in length**  **after filtering** | **Number of length conserved random sequences generated** | **Number of terminal residue conserved random sequences** | **Number of column random sequences generated** |
| --- | --- | --- | --- | --- | --- | --- | --- | --- |
| Mammal | *Homo sapiens*  (GCA_000001405.27) | Reference | GRCh38.p12 | 19,961 | 16,384 | 163,840 | 327,680 | 200,000 |
| Insect | *Drosophila melanogaster*  (GCF_000001215.4) | Reference | 7227 | 30,482 | 24,799 | 247,990 | 495,980 | 200,000 |
| Worm | *Caenorhabditis elegans*  (GCF_000002985.6) | Reference | Bristol N2 | 28,146 | 21,187 | 211,870 | 423,740 | 200,000 |
| Fungi | *Saccharomyces cerevisiae* | Reference | S288C | 5848 | 4772 | 47,720 | 95,440 | 200,000 |
| Fungi | *Aspergillus oryzae*  (GCF_000184455.2) | Representative | RIB40 | 12,074 | 9830 | 98,300 | 196,660 | 200,000 |
| Fungi | *Neurospora crassa*  (GCF_000182925.2) | Representative | OR74A | 10,812 | 8899 | 88990 | 177,980 | 200,000 |
| Bacteria | *Bacillus subtilis*  (GCF_000009045.1) | Reference | str.168 | 4174 | 2588 | 25,880 | 51,760 | 200,000 |
| Bacteria | *Escherichia coli*  (GCF_000005845.2) | Reference | K-12 substr. MG1655 | 4064 | 2838 | 28,380 | 56,760 | 200,000 |
| Bacteria | *Deinococcus radiodurans*  (GCF_000008565.1) | Reference | R1 | 3167 | 2080 | 20,800 | 41,600 | 200,000 |
| Archaea | *Methanosarcina mazei*  (GCF_000970205.1) | Representative | S-6 | 3338 | 2063 | 20,630 | 41,260 | 200,000 |
| Archaea | *Haloferax volcanii*  (GCF_000025685.1) | Representative | DS2 | 3827 | 2410 | 24,100 | 48,200 | 200,000 |
| Archaea | *Thermococcus gammatolerans*  (GCF_000022365.1) | Reference | EJ3 | 2117 | 1346 | 13,460 | 26,920 | 200,000 |
